# Anti-CRISPR-based biosensors in the yeast *S. cerevisiae*

**DOI:** 10.1186/s13036-018-0101-z

**Published:** 2018-08-01

**Authors:** Jing Li, Zengliang Xu, Aleksandr Chupalov, Mario Andrea Marchisio

**Affiliations:** School of Life Science and Technology, 2 Yikuang Street, Nan Gang DistrictHarbin, 150080 People’s Republic of China

**Keywords:** Synthetic biology, Biosensors, Guide RNA, dSpCas9, Anti-CRISPR

## Abstract

**Background:**

Anti-CRISPR proteins are expressed by phages as a reaction to the bacterial CRISPR–Cas defense system. Recently, the structures of anti-CRISPR proteins have been determined, and their diverse functions have been clarified. Anti-CRISPR proteins such as LmAcrIIA2 and LmAcrIIA4 interact with the SpCas9:gRNA system and occlude the protospacer adjacent motif (PAM) recognition site, thereby preventing SpCas9:gRNA from binding to the DNA. Hence, anti-CRISPR proteins represent a powerful means to control and modulate the activity of SpCas9 and its nuclease-deficient version dSpCas9. LmAcrIIA2 and LmAcrIIA4 have been shown to be efficient inhibitors of SpCas9 in *Escherichia coli*, *Saccharomyces cerevisiae*, and mammalian cells. To date, there have been no reports of anti-CRISPR-based synthetic gene circuits engineered into yeast cells.

**Results:**

We constructed in the yeast *S. cerevisiae* synthetic biosensors based on the anti-CRISPR–dSpCas9:gRNA interaction. Upon induction with galactose or *β*-estradiol, anti-CRISPR proteins (LmAcrIIA4, LmAcrIIA2, and StAcrIIA5) produced an enhancement in fluorescence expression by preventing the dSpCas9–Mxi1:gRNA complex from binding to the DNA. We found that LmAcrIIA2 performed as well as LmAcrIIA4 in *S. cerevisiae*, whereas StAcrIIA5, which had previously been tested in bacteria only, had non-negligible negative effects on yeast cell growth. The efficiency of anti-CRISPR-based biosensors was strongly dependent on the means by which the guide RNAs were produced. The best performance, as measured by the increase in fluorescence, was achieved using a “ribozyme–gRNA–ribozyme” expression cassette under the control of the yeast constitutive *ADH1* promoter.

**Conclusions:**

This work demonstrates that anti-CRISPR proteins are effective dSpCas9 suppressors in yeast cells. In particular, LmAcrIIA2 and LmAcrIIA4 could be employed as new components of yeast synthetic gene circuits.

**Electronic supplementary material:**

The online version of this article (10.1186/s13036-018-0101-z) contains supplementary material, which is available to authorized users.

## Background

Anti-CRISPR proteins are used by phages to neutralize the CRISPR–Cas system, a component of the bacterial (and archeal) immune system [25]. Anti-CRISPR proteins bind to the Cas:CRISPR RNA (crRNA) complex and prevent it from binding to and/or cleaving the targeted sequence on the DNA [3, 18]. Among the various anti-CRISPR proteins studied so far, the AcrIIA family has attracted particular interest, as some of its members have been proven to inhibit the type II-A CRISPR–Cas9 system, which is widely used in biotechnology and synthetic biology applications [22].

Rauch et al. [30] identified four anti-CRISPR proteins in the bacterium *Listeria monocytogenes*, referred to as LmAcrIIA1–LmAcrIIA4. LmAcrIIA2 and LmAcrIIA4 were shown to block the activity of *Streptococcus pyogenes* Cas9 (SpCas9) in human cells and its nuclease-deficient version (dSpCas9) in *Escherichia coli* cells. LmAcrIIA4 exhibited better performance than LmAcrIIA2 in both organisms. In *E. coli*, Rauch and co-authors put the synthesis of a red fluorescent protein (RFP) under the negative control of dSpCas9. LmAcrIIA4 was able to almost completely re-establish full RFP expression, whereas with LmAcrIIA2 the red fluorescence level reached only about 25% of that in the absence of a guide RNA (gRNA) targeting RFP. In re-engineered human cells, a green fluorescent protein gene was cleaved by SpCas9. Here, LmAcrIIA4 only slightly outperformed LmAcrIIA2 in inhibiting SpCas9.

Subsequent works [8, 33, 36] reported that both LmAcrIIA2 and LmAcrIIA4 could achieve repression of SpCas9:crRNA activity by mimicking the protospacer adjacent motif (PAM; NGG) recognized by the SpCas9:crRNA complex. These anti-CRISPR proteins occupy the SpCas9 PAM binding site and prevent recognition of the DNA. Interestingly, this takes place only when SpCas9 is associated with crRNA.

More recently, during the peer-review process for this work, Basgall et al. [1] successfully used both LmAcrIIA2 and LmAcrIIA4 as inhibitors of SpCas9 in a gene drive system for the yeast *Saccharomyces cerevisiae*.

Another AcrAII protein was identified in *Streptococcus thermophilus* and termed StAcrIIA5. This anti-CRISPR protein could inhibit SpCas9 in *Lactococcus lactis* but not in *E. coli* [19]. It was not tested in eukaryotic cells or, in general, on dSpCas9.

We evaluated the functions of these three anti-CRISPR proteins in the yeast *S. cerevisiae*. Each of them acted on a yeast codon-optimized version of dSpCas9 (fused to a nuclear localization sequence–NLS) [10] targeting a transcription unit that encoded the yeast enhanced green fluorescent protein (yEGFP [32]). Specifically, we constructed galactose- and *β*-estradiol-biosensing devices (buffer, or YES, gates in logic terms) based on dSpCas9:gRNA–anti-CRIPSR interactions. YES gates are essential components in the design of complex gene digital circuits. They transduce input signals (chemicals) into proteins or small RNAs. In this way, the inputs are transmitted through the internal gates to the output gates that return fluorescence or activate/repress a cellular pathway [24]. Digital circuits have several applications in synthetic biology, from bio-computing [2] to pollutant detection and disease recognition [23]. We chose galactose and *β*-estradiol for a proof-of-concept experiment, since the former activates *GAL1* promoter directly, while the latter exerts its action on the anti-CRISPR protein upon its fusion to a proper hormone binding domain (see below). Hence, these two chemicals did not require our circuits to include more than four genes, as there are genetically modified auxotrophic markers in the CEN.PK2-1C yeast strain used in this work (see Methods).

In order to construct the buffer gates, we first evaluated the efficiency of transcriptional repression by means of dSpCas9:gRNA. In particular, we used different modes of gRNA expression, namely via the RNA polymerase III-dependent promoters *SNR52* [7] and *RPR1* [10] (both on integrative and multicopy plasmids), and using the RGR cassette HH ribozyme–gRNA–HDV ribozyme (HH, hammerhead; HDV, hepatitis delta virus) under the RNA polymerase II-dependent *ADH1* promoter [12]. Moreover, we tested both the bare dSpCas9 and dSpCas9 fused to the repression domain Mxi1 [11, 15].

After identifying the best solution for repressing the production of yEGFP from the synthetic promoter Tsynth8_pCYC1noTATA [35] employed in our circuits, we constructed several small networks (four genes overall, as mentioned above), each containing one of the anti-CRISPR proteins LmAcrIIA2, LmAcrIIA4, and StAcrIIA5. They were expressed upon induction with galactose. Both LmAcrIIA2 and LmAcrIIA4 worked well in *S. cerevisiae*, resulting in a significant increase (more than three-fold) in cell fluorescence via inhibiting the action of dSpCas9:gRNA on Tsynth8_pCYC1noTATA. Finally, we constructed two hormone biosensors by fusing LmAcrIIA2 to the hormone binding domain of the human estrogen receptor, HBD(ER) [20]. In the presence of 1 *μM* *β*-estradiol, both circuits were able to prompt an approximately two-fold enhancement in fluorescence.

## Results and discussion

The output of our biosensing circuits, which were based on dSpCas9:gRNA–AcrAII (anti-CRISPR) interactions, was yEGFP. This reporter protein is expressed by the synthetic promoter Tsynth8_pCYC1noTATA, produced by fusing a synthetic terminator (Tsynth8 [5]) to the minimal *CYC1* promoter stripped of its TATA boxes (pCYC1noTATA [35]). Thus, the efficiency element of Tsynth8 became the TATA box of the synthetic promoter. Tsynth8_pCYC1noTATA is down-regulated by the complex dSpCas9:gRNA. The gRNA contained a 20-nt spacer ATAAACTCATTTACTTATGT that overlapped the promoter TATA box. We verified with CRISPRdirect [27] that this 20-mer is absent from the yeast *S. cerevisiae* genome; this was as expected, since it is a portion of a synthetic terminator. Moreover, the spacer seed region (i.e., the last 12 nucleotides [7]) had no perfect match in the budding yeast genome either.

Anti-CRISPR proteins, which interact with and inhibit the dSpCas9:gRNA complex, are produced upon induction with galactose or translocate into the nucleus upon induction with *β*-estradiol. We considered a working biosensor to be any circuit where the fluorescence level expressed in the presence of the input signal was significantly different, in statistical terms, from that measured without the input (two-sided Welch’s *t*-test, *p*-value < 0.05).

### dSpCas9:gRNA-based transcriptional control in *S. cerevisiae*

The first step in the construction of our biosensing devices was to test the efficiency of the dSpCas9:gRNA system in repressing transcription from the Tsynth8_pCYC1noTATA synthetic promoter.

So far, two main strategies have been adopted for gRNA expression: (1) using an RNA polymerase III-dependent promoter such as pRPR1 (and its corresponding terminator, RPR1t [10]) or pSNR52 (together with the *SUP4* terminator, SUP4t [7]); and (2) flanking the gRNA with two ribozymes [11, 12] (RGR cassette). In the latter case, an RNA polymerase II-type promoter leads to the production of long mRNA chains. Upon mRNA formation, the two ribozymes autocleave and release a gRNA molecule.

dSpCas9 was used either in its natural conformation (the sequence was usually codon optimized for expression in *S. cerevisiae*) or fused to the repression domain Mxi1. The presence of Mxi1 has been reported to enhance the performance of dSpCas9 in transcription regulation [15].

Under the assumption that RNA polymerase III-dependent promoters are weak [11], transcription units containing RNA polymerase III elements for gRNA synthesis were generally placed into multicopy (or centromeric [6, 34]) plasmids. This guaranteed the expression of gRNA in a reasonably high copy number, but required cells to be grown in selective media. By contrast, the RGR cassette was placed into integrative plasmids downstream of strong RNA polymerase II-type promoters (such as pADH1 [12]). The RGR secures circuit stability (owing to its integration into the genome) and should provide gRNA in large amounts. However, the choice of promoter and terminator at the edge of the RGR are limited to those that allow a proper folding of both HH and HDV ribozymes.

Since the efficiency of transcription regulation via dSpCas9:gRNA has been shown to be highly context dependent in general [34], we decided to build eight inducible three-gene circuits (NOT gates) to assess the performance of the above-described designs and test new ones with our synthetic promoter.

**Fig. 1:**
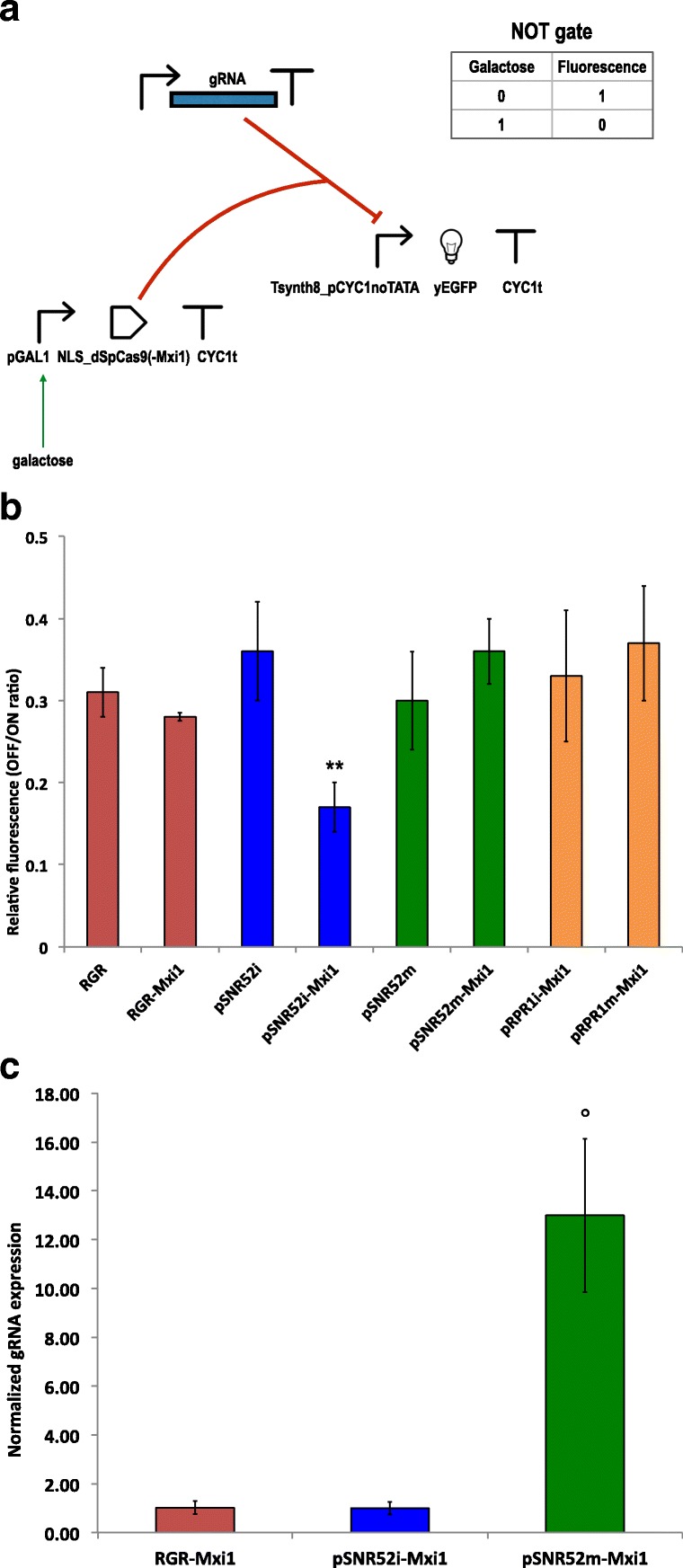
NOT gates used to test the action of dSpCas9(-Mxi1):gRNA on the synthetic promoter Tsynth8_pCYC1noTATA. **a** Circuit scheme. The guide RNA is expressed either via an RGR cassette or an RNA polymerase III-dependent promoter (pSNR52 or pRPR1). Galactose induces the synthesis of dSpCas9(-Mxi1). **b** Ratio between the NOT gate fluorescence level in the presence (OFF state) and absence (ON state) of galactose. Each gate is labelled with the expression system for the guide RNA (RGR cassette–red color; pSNR52i: pSNR52-gRNA-SUP4t on an integrative plasmid–blue; pSNR52m: pSNR52-gRNA-SUP4t on a multicopy plasmid–green; pRPR1i: pRPR1-gRNA-RPR1t on an integrative plasmid–orange; pRPR1m: pRPR1-gRNA-RPR1t on a multicopy plasmid–orange) followed by Mxi1 when this repression domain was attached to dSpCas9. The highest fluorescence repression was obtained by expressing the guide RNA via the *SNR52* promoter on an integrative plasmid together with dSpCas9 fused to Mxi1 (pSNR52i-Mxi1). This NOT gate configuration clearly outperforms the other seven (the “ ∗∗” symbol on top of the corresponding bar indicates a statistically significant difference from all the other constructs–two-sided Welch’s t-test, p-value < 0.05). Each relative fluorescence level is the mean value obtained from 3 up to 6 independent experiments (i.e. carried out in different days). Further statistical considerations are reported in Additional file 1: Table S1 and Figure S1. **c** Normalized gRNA expression from the three NOT gate schemes selected to build the anti-CRISPR-based biosensors. For each gate, the relative amount of gRNA with respect to the mRNA produced by the *ACT*1 gene was first calculated (mean value from three replicates during a single experiment– 12.5 *ng* of cDNA were used). Then, gRNA relative expressions were normalized to the value obtained for pSNR52i-Mxi1. Error bars were determined on the normalized values via the error propagation formula. The *ADH1* promoter present in the RGR-Mxi1 configuration appears to drive the synthesis of as much gRNA (1.02 ±0.26) as the RNA polymerase III-dependent *SNR52* promoter on an integrative plasmid i.e. the reference strain pSNR52i-Mxi1 to which the value 1.00 (± 0.26) is assigned (two-sided Welch’s t-test, *p*-value =0.96). In contrast, pSNR52m-Mxi1–where pSNR52 is placed on a multicopy plasmid–expresses 12.99 (± 3.15)-fold more gRNA than pSNR52i-Mxi1 (the “ ∘” symbol points out a statistically significant difference with respect to the reference gate–two-sided Welch’s t-test, *p*-value < 0.05). Thus, gRNA expression is not directly correlated with NOT gate efficiency since lower gRNA levels–as in pSNR52i-Mxi1 and RGR-Mxi1–correspond to higher fluorescence repression

Each of these NOT gates contained a transcription unit that expressed the guide RNA constitutively. Three variants of this transcription unit were constructed: pADH1–RGR–ADH1t (always into an integrative plasmid), pRPR1–gRNA–RPR1t, and pSNR52–gRNA–SUP4t. The two units based on RNA polymerase III-dependent promoters and terminators were placed into both multicopy and integrative plasmids. Synthesis of dSpCas9 or dSpCas–Mxi1 was induced by galactose. Finally, the circuit output (fluorescence) was expressed under Tsynth8_pCYC1noTATA (see Fig. 1a).

With the exception of pRPR1–gRNA–RPR1t, which was used with dSpCas9–Mxi1 only, we tested the working of each transcription unit for gRNA expression together with both the bare dSpCas9 and that fused to the Mxi1 repression domain. As shown in Fig. 1b, the strongest fluorescence repression (83%) was observed in the presence of dSpCas9–Mxi1 and the RNA polymerase III-dependent transcription unit pSNR52–gRNA–SUP4t placed on an integrative plasmid (concisely, we refer to this configuration as pSNR52i–Mxi1). The other circuit configurations showed repression varying from 63% (pRPR1m–Mxi1, i.e., pRPR1–gRNA–RPR1t on a multicopy plasmid and dSpCas9–Mxi1) to 72% (RGR–Mxi1, i.e., the RGR cassette and dSpCas9–Mxi1). In statistical terms, pSNR52i–Mxi1 was significantly different from each of the other seven NOT gates (see Additional file 1: Figure S1). Moreover, the only other statistically significant difference was between RGR–Mxi1 and pSNR52m–Mxi1. The latter circuit employs pSNR52 on a multicopy plasmid for the synthesis of the gRNA.

As pointed out above, RNA polymerase III-dependent promoters are considered to be weak promoters. Hence, it was assumed that transcription units such as pSNR52–gRNA–SUP4t or pRPR1–gRNA–RPR1t would not express enough gRNA molecules upon integration into the *S. cerevisiae* genome to result in a working CRISPR–(d)SpCas9 system. To the best of our knowledge, however, this assumption has never been proven. In fact, our results (Fig. 1b) indicate that the quantity of gRNA is not a limiting factor, at least for certain kinds of synthetic gene circuits such as NOT gates.

For the construction of biosensors based on anti-CRISPR proteins we selected three configurations. First, we chose the design that outperformed all the others in terms of repression of fluorescence, i.e., pSNR52i–Mxi1. Then, we picked two other schemes that employed dSpCas9–Mxi1: RGR–Mxi1 (the second-best NOT gate in terms of mean relative fluorescence) and the lower-performing pSNR52m–Mxi1 (64% fluorescence repression; see Fig. 1b). We made this choice so as to examine the possible influence of the gRNA expression system (the integrative/multicopy plasmid carrying the pSNR52 promoter, or the RGR cassette) on the working of the anti-CRISPR-containing circuits. It should be noted that there were statistically significant differences in the relative fluorescence of these three NOT gates.

We carried out real-time quantitative polymerase chain reaction (RT-qPCR) [6, 31] to quantify the relative amount of gRNA expressed by each of these three NOT gates. *ACT*1 was used a reference gene in these measurements. pSNR52m–Mxi1, the least efficient among these circuits, expressed almost 13-fold more gRNA than the other two gates (Fig. 1c). Moreover, pSNR52i–Mxi1 and RGR–Mxi1 produced roughly the same quantities of gRNA. Nevertheless, the fluorescence repression achieved with pSNR52i–Mxi1 was clearly higher than that achieved with RGR–Mxi1. Taken together, these results confirmed that the working of our NOT gates was independent of the gRNA expression level.

In a biosensing device, the production (or the translocation into the nucleus) of anti-CRISPR proteins is triggered by an input signal, whereas both gRNAs and dSpCas9–Mxi1 are constitutively expressed. For this reason, we tested the efficiency of three sub-circuits based on the chosen NOT gate configurations. These differed from the circuit shown in Fig. 1a in terms of the promoter controlling dSpCas9–Mxi1 expression: the *GPD* promoter (pGPD) was used instead of pGAL1 (see Additional file 1: Figure S2A). In each case, we compared the fluorescence level (low) of the whole three-gene circuit with that of a two-gene circuit lacking either dSpCas9–Mxi1 or the gRNA. With respect to the NOT gates, we did not detect any difference in the repression level associated with the three-gene circuit hosting a multicopy plasmid (64%). However, the configuration with the RGR cassette performed better than that with pSNR52 in an integrative plasmid: 87% of fluorescence repression versus 75% (see Additional file 1: Figure S2B and Table S2). We attributed this discrepancy with respect to the NOT gates mainly to the fact that we did not use the same two-gene control circuit for each of the three sub-circuits. In particular, the control circuit for the RGR cassette did not contain the transcription unit for the synthesis of dSpCas9–Mxi1 under the strong *GPD* promoter. As a consequence, the fluorescence level of the RGR control was higher than that of the other two-gene devices, which lacked the gRNA expression plasmid. RT-qPCR experiments confirmed the results obtained for the NOT gates, since the circuits associated with the highest fluorescence repression had the lowest gRNA expression (Additional file 1: Figure S2C). This further underlines that the gRNA amount is not a limiting factor in achieving a dSpCas9:gRNA complex concentration sufficient to repress transcription from the synthetic promoter Tsynth8_pCYC1noTATA.

### Biosensors based on anti-CRISPR proteins

In order to construct biosensors that exploit the inhibition of the dSpCas9–Mxi1:gRNA system by anti-CRISPR proteins, we extended the three sub-circuits described above with a further transcription unit encoding one of LmAcrIIA2, LmAcrIIA4 [30], and StAcrIIA5 [19]. LmAcrIIA2 and LmAcrIIA4 were shown to inhibit both SpCas9 and its nuclease-deficient version, the former in eukaryotic cells and the latter in bacteria. StAcrIIA5 was proven to suppress DNA cleavage from SpCas9 into bacterial cells. However, the way StAcrIIA5 interacts with SpCas9 is unknown. Notably, some anti-CRISPR proteins, such as AcrIIC1, hinder Cas9 DNA cleavage not by preventing Cas9 from binding to the target DNA sequence (i.e., by occluding the Cas9 PAM recognition site) but by inactivating Cas9 nuclease activity upon binding to the Cas9 HNH nuclease domain [18]. In other words, AcrIIC1 turns Cas9 into a dCas9. Therefore, the fact that an anti-CRISPR protein, such as StAcrIIA5, prevents SpCas9 from cutting the DNA does not necessarily imply that the same protein impedes dSpCas9 from binding to the DNA.

**Fig. 2:**
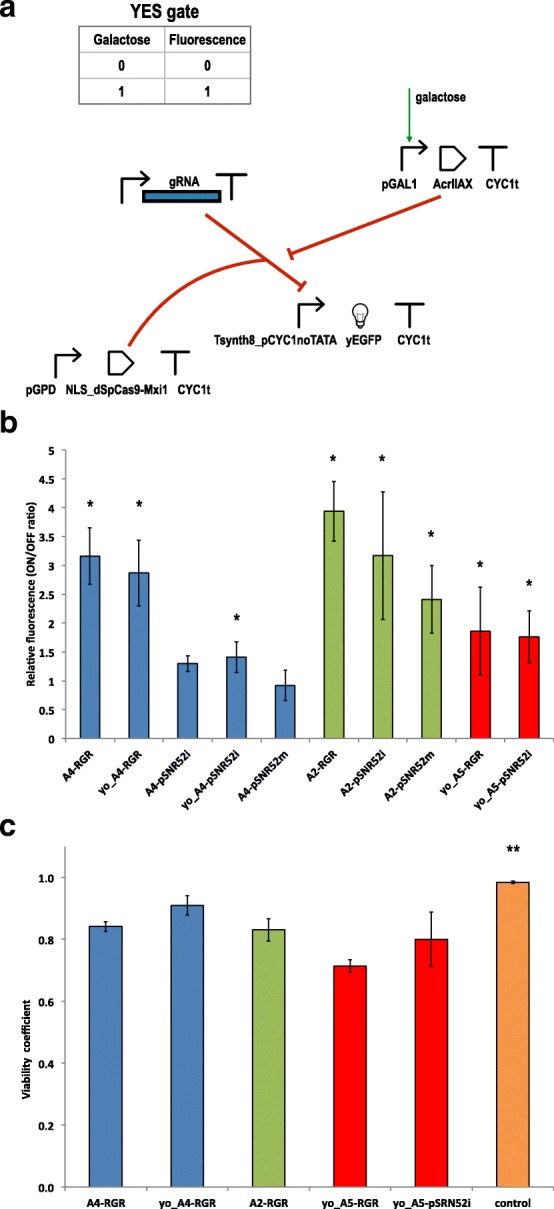
Galactose biosensors based on anti-CRISPR proteins. **a** Upon induction with galactose, an anti-CRISPR protein of the AcrIIA family is produced. An increase in fluorescence is detected if the anti-CRISPR is able to prevent the dSpCas9-Mxi1:gRNA system from binding the Tsynth8_pCYC1noTATA synthetic promoter. **b** Galactose biosensor performance. Main values of the ON/OFF ratio were calculated over at least 3 independent experiments for each circuit. The symbol “*” indicates a working biosensor i.e. there is a statistically significant difference between the mean fluorescence of the ON and OFF state (two-sided Welch’s t-test, *p*-value < 0.05). A detailed statistical comparison of the relative fluorescence of these ten anti-CRIPSR-based constructs is given in Additional file 1: Figure S3. **c** Viability test. The viability coefficients of five YES gates presenting slow growth rate underline the presence of only moderate toxic effects. The control strain (A5-RGR grown in glucose-supplied synthetic medium) is associated with a viability coefficient, equal to 0.98, significantly higher in statistical terms than all the other viability coefficients calculated in this test (as denoted by the symbol “**”). A further statistical analysis on these results is reported in Additional file 1: Figure S8

We placed the anti-CRISPR coding sequence downstream of the *GAL1* promoter. Hence, the presence of galactose in the cell-growth solution should induce an increase in the fluorescence expressed by our circuits (buffer, or YES, gates; see Fig. 2a) to a level significantly different, in statistical terms, from that expressed in the presence of glucose.

We started with LmAcrIIA4 because this protein is known to be a strong SpCas9 inhibitor in human cells [30]. Circuit performance was assessed by calculating the ratio between the fluorescence levels in the presence and absence of galactose (see Fig. 2b and Additional file 1: Table S3). LmAcrIIA4 performed well only when paired with the RGR cassette for gRNA expression. The circuit hosting the original sequence of LmAcrIIA4 together with the RGR (A4-RGR) showed an almost 3.2-fold gain in fluorescence upon induction with galactose. We obtained a statistically equivalent result by using an LmAcrIIA4 sequence codon optimized for expression in *S. cerevisiae* (yo_LmAcrIIA4. YES gate: yo_A4-RGR). When the RGR cassette was replaced with the transcription unit pSNR52–gRNA–SUP4t (on both the integrative and the multicopy plasmid), only one of the three circuit variants functioned correctly, i.e., showed a statistically significant difference between fluorescence levels with and without galactose. This configuration made use of yo_LmAcrIIA4 and pSNR52 on an integrative plasmid for gRNA synthesis (yo_A4-pSNR52i). The corresponding gain in fluorescence, however, was modest (1.41-fold).

**Fig. 3:**
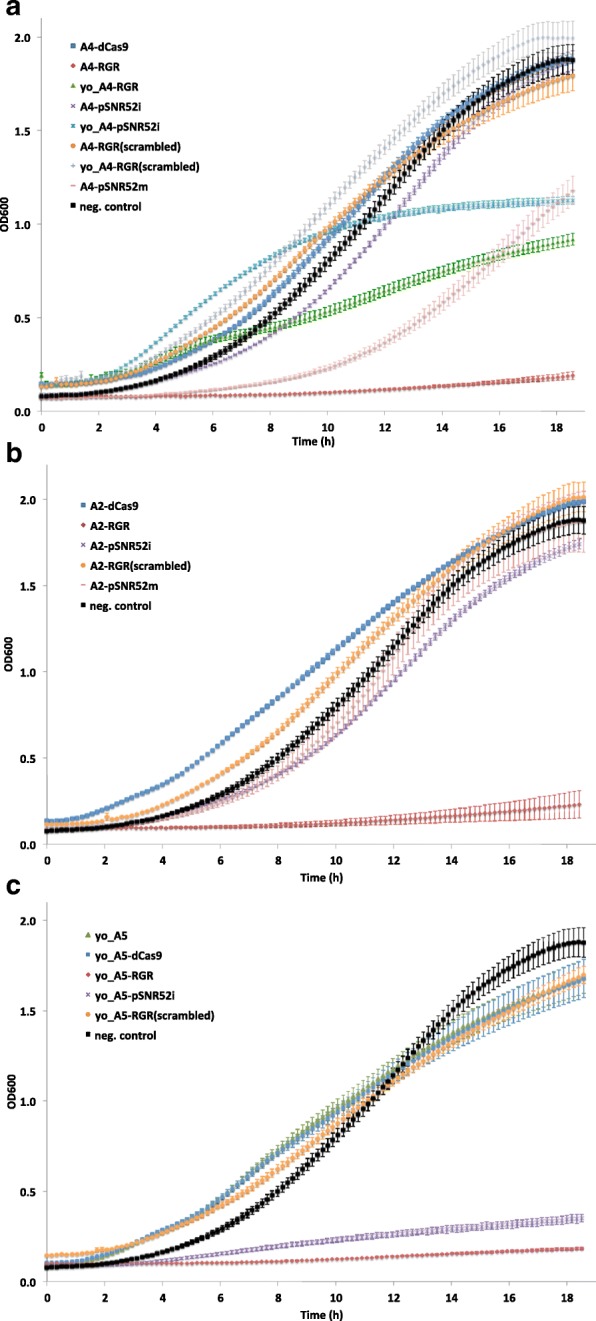
Growth curves in synthetic medium supplied with 2% galactose. **a** LmAcrIIA4-hosting circuits. The three working biosensors (A4-RGR, yo_A4-RGR, and yo_A4-pSNR52i) grew more slowly than byMM234, the negative control that expresses only yEGFP. By removing the gRNA from the circuit (dCas9-A4) or substituting it with a scrambled one with no match along the yeast genome (A4-RGR(scrambled) and yo_A4-RGR(scrambled)) a quick growth was re-established. **b** Biosensors based on LmAcrIIA2 confirmed that the RGR cassette is responsible for a slow growth when gRNAs targeting Tsynth8_pCYC1noTATA are expressed. In the presence of a scrambled gRNA (A2-RGR(scrambled)) as well as in the absence of any gRNA molecules (A2-dCas9) cell growth rate appeared even faster than that of the negative control. pSNR52 both on integrative (A2-pSNR52i) and multicopy (A2-pSNR52m) plasmid did not provoke drastic changes in cell growth either. **c** Both biosensors containing yo_StAcrIIA5 (yo_A5-RGR and yo_A5-pSNR52i) grew extremely slowly. However, the protein yo_StAcrIIA5 on its own has no negative effects on yeast cell growth as witnessed by the fast growth rate of the three control circuits (yo_A5, yo_A5-dCas9, and yo_A5-RGR(scrambled). Every average OD600 value was calculated on three replicates (single experiment)

The reason for the malfunctioning of the galactose biosensors hosting LmAcrIIA4 and a gRNA expression system based on the *SNR52* promoter (A4-pSNR52i and A4-pSNR52m) is not clear. We traced the growth curves over more than 18 h in synthetic medium containing galactose for the five strains described above, in order to see whether the non-working biosensors manifested any growth problems. With respect to our negative control (byMM234, i.e., the strain expressing yEGFP under Tsynth8_pCYC1noTATA without any transcription regulation) A4-pSNR52m showed an evident reduction in the growth rate, whereas A4-pSNR52i grew more slowly in a much less remarkable way and only between approximately 5 and 15 h. These two strains, however, grew substantially faster than A4-RGR (an efficient biosensor) from the early stages of the optical density measurements, and appeared to grow more rapidly than the other two working biosensors (yo_A4-RGR and yo_A4-pSNR52i) after about 12 h (A4-pSNR52i) or towards the end of the experiment (A4-pSNR52m). The usage of yo_LmAcrIIA4 had the opposite effects on the growth of synthetic yeast cells: it sped up the growth of the RGR-containing strain (yo_A4-RGR), whereas it slowed down (after about 12 h) the growth of the pSNR52i-based strain (yo_A4-pSNR52i; see Fig. 3a). Interestingly, the two strains carrying the RGR cassette together with A4-pSNR52m also appeared to grow more slowly than the others in glucose-containing synthetic medium (Additional file 1: Figure S4A).

A faster growth rate (though only after about 11 h) with respect to these five strains was achieved by modifying byMM234 with the insertion of LmAcrIIA4 and dSpCas9–Mxi1 only, i.e., without any transcription unit for gRNA synthesis. In this configuration (termed A4-dCas9; Fig. 3a), LmAcrIIA4 could not bind to dSpCas9–Mxi1. We obtained an almost identical growth curve by expressing a “scrambled” gRNA – via the RGR cassette – in the presence of LmAcrIIA4 and dSpCas9–Mxi1 (A4-RGR(scrambled)). The scrambled gRNA contained a 20-nt spacer that targeted the LacI bacterial repressor and had no match in the *S. cerevisiae* genome. Control experiments on the three-gene sub-circuits lacking LmAcrIIA4 confirmed that the RGR cassette *per se* had no negative influence on yeast cell growth but provoked a slowdown of the growth rate only when producing gRNA molecules able to bind to their target on the DNA (Additional file 1: Figure S5). However, it is not clear why this effect was less evident when the gRNA was expressed by the *SNR52* promoter placed on a multicopy plasmid and almost absent when an integrative plasmid was used instead.

We next constructed galactose-sensing devices based on LmAcrIIA2 in order to see whether we could observe any of the features present in the LmAcrIIA4-based circuits.

LmAcrIIA2 has been reported to have lower efficiency than LmAcrIIA4, in both bacteria and human cells. In our experiments, we did not observe a clear gap between these proteins; thus, we can assert that, in *S. cerevisiae*, LmAcrIIA2 and LmAcrIIA4 have comparable performance on dSpCas9:gRNA. Each of the three galactose sensors based on LmAcrIIA2 returned an average gain in fluorescence greater than two-fold upon induction with galactose. However, the uncertainty on the relative fluorescence values were also rather large, especially when the gRNA was expressed via pSNR52 on an integrative plasmid (A2-pSNR52i; see Fig. 2b and Additional file 1: Table S3). Similar to the circuits realized with LmAcrIIA4, the best performance was observed with the biosensor hosting the RGR cassette (A2-RGR), a 3.94-fold average gain. This circuit was significantly different from all the other galactose-biosensing networks we constructed, with the exception of A4-RGR and A2-pSNR52i (it should be noted, though, that A2-pSNR52i was statistically indistinguishable from any of our working biosensors; see Additional file 1: Figure S3). In contrast to LmAcrIIA4, we managed to build a working biosensor based on LmAcrIIA2 and the gRNAs expressed via pSNR52 on a multicopy plasmid, A2-pSNR52m, that gave a 2.41-fold average gain in fluorescence.

The growth curves in synthetic medium supplied with 2% galactose reproduced only partially the trend observed in the circuits hosting LmAcrII4. The slowest growth rate was detected, again, in the presence of the RGR cassette for the production of gRNAs able to bind to the promoter Tsynth8_pCYC1noTATA (A2-RGR). However, in contrast to the LmAcrII4-based biosensors, the expression of gRNAs from multicopy plasmids containing pSNR52 (A2-pSNR52m) had almost no influence on yeast cell growth. Indeed, the A2-pSNR52m growth curve almost completely overlapped that of byMM234 (Fig. 3b), while pSNR52 on an integrative plasmid (A2-pSNR52i) induced only a small slowdown in cell growth rate. These two circuits also had little effect on cell growth in glucose-containing solution (Additional file 1: Figure S4B).

Finally, we constructed two biosensors by integrating into the genome of *S. cerevisiae* a yeast codon-optimized version of the anti-CRISPR protein StAcrIIA5 (yo_StActIIA5) together with either the RGR cassette (yo_A5-RGR) or the pSNR52–gRNA–SUP4 transcription unit (yo_A5-pSNR52i) for gRNA expression. For both circuits, the main values of fluorescence expressed in the presence of galactose were statistically significantly different from those obtained in the absence of galactose, and the fluorescence gain was close to two-fold (Fig. 2b); however, the cell populations did not show well-defined shapes in the fluorescence activated cell sorting (FACS) dot plots (Additional file 1: Figure S6). Furthermore, both yo_A5-RGR and yo_A5-pSNR52i showed remarkably slow growth rates in synthetic medium containing galactose, as shown in Fig. 3c (in the presence of glucose, only the growth curve of yo_A5-RGR deviated considerably from that of the negative control; see Additional file 1: Figure S4C). To determine whether yo_StActIIA5 was less tolerated by *S. cerevisiae* than (yo_)LmAcrIIA4 and LmAcrIIA2, we built three control circuits in which byMM234 was modified with the expression of (1) the sole yo_StActIIA5 (yo_A5), (2) yo_StActIIA5 plus dSpCas9–Mxi1 (yo_A5-dCas9), or (3) yo_StActIIA5 together with dSpCas9–Mxi1 and an RGR cassette synthesizing the scrambled gRNA described above (yo_A5-RGR(scrambled)). In galactose-containing solution, these three circuits showed almost indistinguishable growth curves, with shapes similar to that of the negative control (Fig. 3c). These results, which are consistent with our measurements on analogous circuits involving (yo)_LmAcrIIA4 and LmAcrIIA2, indicate that the anti-CRISPR protein yo_StActIIA5 is not toxic *per se* to yeast cells. However, mild toxicity effects might be present when the system yo_AcrIIA5–dSpCas9:gRNA interacts with the DNA and causes the irregular yeast population shapes that emerged during the FACS experiments. To verify this hypothesis, we ran a viability test on the strains yo_A5-RGR and yo_A5-pSNR52i. For comparison with the other circuits that had growth curves significantly lower than that of byMM234 in galactose-containing medium (Additional file 1: Figure S7), we also calculated the viability coefficients for A4-RGR, yo_A4-RGR, and A2-RGR. As a negative control, we took yo_A5-RGR grown in glucose-containing solution (Fig. 2c); yo_A5-RGR gave the lowest mean value of the viability coefficient (0.71), a statistically significant difference compared with all the other circuits except for yo_A5-pSNR52i (Additional file 1: Figure S8 and Table S4). The high relative error (11.25*%*) on the viability coefficient of yo_A5-pSNR52i caused this measurement to be statistically indistinguishable from all the others (apart from the negative control). This result was consistent with the considerable variability in cell size highlighted by the FACS dot plot on yo_A5-pSNR52i. A4-RGR and A2-RGR gave comparable results (0.84 and 0.83, respectively), significantly higher than the value of yo_A5-RGR and lower than that of yo_A4-RGR (0.91), the circuit with the highest growth rate in galactose-containing medium. Overall, the viability test showed that only moderate toxicity effects, which did not preclude the working of our YES gates, were induced to different extents by the interactions of diverse anti-CRISPR proteins with the same dSpCas9:gRNA complex and target DNA sequence.

In summary, LmAcrIIA2 and LmAcrIIA4 have similar performance in *S. cerevisiae* as inhibitors of transcriptional repression by dSpCas9–Mxi1:gRNA. In our galactose-sensing devices, where the presence of galactose stimulates the production of anti-CRISPR proteins, the highest gain in fluorescence was obtained by expressing the gRNA via an RGR cassette. This configuration provoked a drastic decrease in the growth of yeast cells, without relevant toxicity effects. The use of a yeast codon-optimized version of LmAcrIIA4 together with the RGR cassette speeded up the growth of biosensor-carrying cells, increased (above 0.90) the value of the viability coefficient, and returned an ON/OFF ratio comparable with that of the A4-RGR circuit.

LmAcrIIA2 performed well not only in the presence of the RGR but also when paired with the RNA polymerase III-type *SNR52* promoter on integrative and multicopy plasmids. In both cases, the decrease in cell growth rate was almost negligible.

StAcrIIA5-based biosensors, by contrast, showed lower ON/OFF ratios and a slowdown in the growth curves of cells containing both the RGR and pSNR52–gRNA–SUP4t transcription unit on an integrative plasmid. High variability in cell size was also apparent in the dot plots from FACS experiments. Moreover, the viability coefficient calculated on the yo_A5-RGR circuit was significantly lower than those associated with the LmAcrIIA2- and (yo_)LmAcrIIA4-hosting biosensors.

Overall, LmAcrIIA2 and (yo_)LmAcrIIA4 can be considered as new, reliable components of Boolean gates in *S. cerevisiae*. The reduced cell growth (in the presence of the RGR cassette) was not detrimental to the biosensor’s performance, nor was it associated with any relevant toxic effects. Hence, we argue that the YES gates here described could be used inside more complex digital circuits to convert an input signal into, for instance, a transcription factor acting on other circuit gates. Moreover, cell growth rates could be modulated either by choosing different expression systems for the gRNAs that, together with LmAcrIIA2, result in high gains in fluorescence, or by using an yeast codon-optimized version of the anti-CRISPR protein, as in the case of LmAcrIIA4. yo_StAcrIIA5, by contrast, showed more significant negative side effects on cell growth, and thus requires further characterization before being employed inside synthetic gene networks in *S. cerevisiae*.

### Biosensors responding to *β*-estradiol

As LmAcrIIA2 performed well with every gRNA expression system, we chose this anti-CRISPR protein to build a further biosensor that responds to *β*-estradiol. We fused LmAcrIIA2 to the HBD(ER) [20], which is known to interact with and be bound by the heat-shock protein Hsp90. As a result, a chimeric protein containing HBD(ER), such as our LmAcrIIA2–HBD(ER), would be sequestered into the cytoplasm by Hsp90. Upon binding to *β*-estradiol, HBD(ER) undergoes a conformational change that prevents any further interactions with Hsp90. Hence, the chimeric protein can migrate into the nucleus. In our case, the presence of *β*-estradiol induced the interaction of LmAcrIIA2–HBD(ER) with dSpCas9–Mxi1:gRNA. So far, in *S. cerevisiae*, HBD(ER) has been used mainly as a means to engineer synthetic transcription factors [20, 21, 26, 28].

**Fig. 4:**
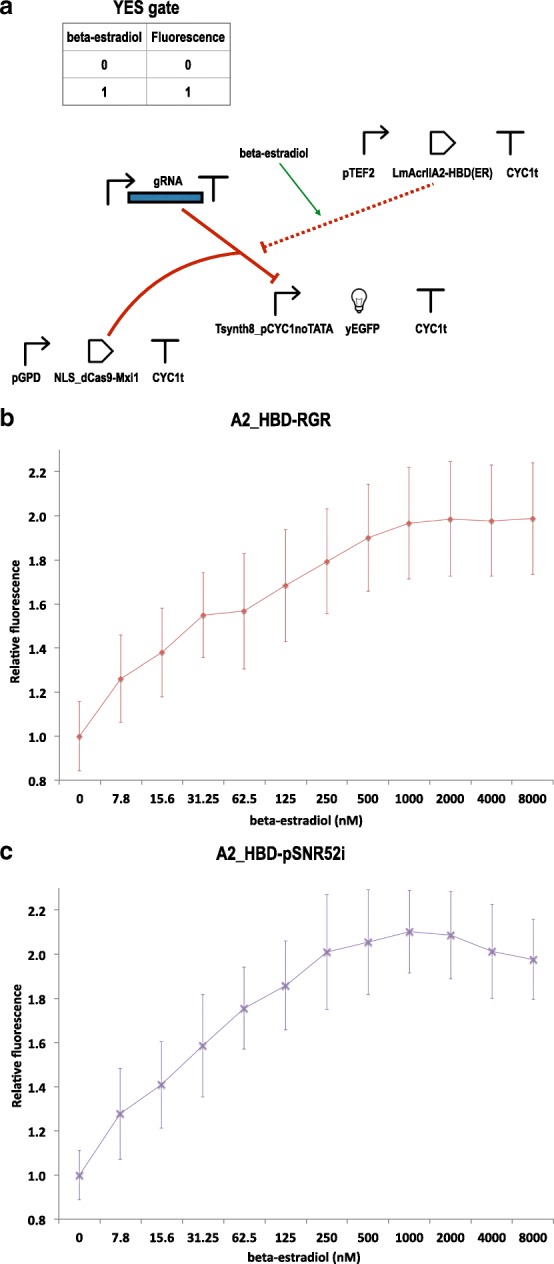
*β*-estradiol sensing circuits. **a** Upon induction with *β*-estradiol the chimeric protein LmAcrIIA2-HDB(ER) enters the nucleus and prevents dSpCas9-Mxi1:gRNA from binding Tsynth8_pCYC1noTATA promoter. Hence, the overall effect of *β*-estradiol is to *activate* mRNA transcription from Tsynth8_pCYC1noTATA, as symbolized by the green arrow. The dashed red line indicates that, in its ground-state configuration, LmAcrIIA2-HDB(ER) is unable to interact with dSpCas9-Mxi1:gRNA due to the binding of Hsp90 to HBD(ER). **b**-**c** Titration curves. The fluorescence gain of the A2_HBD-RGR (**b**) and A2_HBD-pSNR52i (**c**) upon induction with *β*-estradiol is estimated for 11 different hormone concentrations. Each fluorescence value was obtained as the mean of four independent measurements (3 replicates only were available for A2_HBD-RGR at 31.25 *nM* and A2_HBD-pSNR52i at 2 *μM*). The standard deviations of the mean values of relative fluorescence were calculated via the error propagation formula

We constructed two circuits, one expressing the gRNAs via the RGR cassette (A2_HBD-RGR), the other through pSNR52–gRNA–SUP4t on an integrative plasmid (A2_HBD-pSNR52i). As depicted in Fig. 4a, the chimeric protein LmAcrIIA2–HBD(ER) was placed downstream of the yeast *TEF2* promoter (47% as strong as pGPD [35]).

We tested the two circuits at different concentrations of *β*-estradiol, from 7.81 *nM* to 8 *μM*. At each concentration, we calculated the fluorescence expressed by each circuit relative to the fluorescence level detected in the absence of the hormone (Fig. 4b-c). The two circuits had comparable performance. Their steady state, which corresponded to an approximately two-fold gain in fluorescence expression, was reached at 250*nM* *β*-estradiol (2.01 ±0.26) by A2_HBD–pSNR52i and at 1*μM* *β*-estradiol (1.96 ±0.25) by A2_HBD–RGR. In a later attempt to improve the circuit performance, we tried to express LmAcrIIA2–HBD(ER) under pGPD; however, this resulted in the synthesis of too much LmAcrIIA2–HBD(ER), such that a substantial amount of this chimeric protein could enter the nucleus and inhibit dSpCas9–Mxi1:gRNA even in the absence of *β*-estradiol, preventing correct functioning of our circuit (Additional file 1: Figure S9).

Notably, A2_HBD-RGR resulted in a slower growth rate with respect to A2_HBD–pSNR52i, both in the presence and absence of *β*-estradiol, confirming the trend observed with A2-RGR and A2-pSNR52i. However, the slowdown in the growth of A2_HBD–RGR was much less remarkable than that corresponding to A2-RGR. Moreover, the viability coefficients calculated for the two *β*-estradiol sensors were proximal to 1 both in the presence and absence of *β*-estradiol (Additional file 1: Figure S10 and Table S5).

## Conclusions

In this work, we have described a new class of synthetic biosensors in the yeast *S. cerevisiae*, based on the interactions between anti-CRISPR proteins and the dSpCas9–Mxi1:gRNA system. First, we assessed how different dSpCas9:gRNA configurations could repress the synthesis of a reporter protein from a synthetic promoter built in our laboratory. The 20-mer spacer sequence of the gRNA was chosen both to bind in the proximity of the promoter TATA-box (to enhance transcriptional repression) and to avoid off-target matches within the yeast genome. Contrary to the belief that RNA polymerase III-dependent promoters are much weaker than promoters bound by RNA polymerase II and need to be present in a high copy number to assure a reasonable gRNA expression, we achieved the strongest fluorescence repression by inserting the transcription unit pSNR52–gRNA–SUP4t into an integrative plasmid. dSpCas9 was fused to the Mxi1 repression domain and placed downstream of the *GAL1* promoter. Uninduced systems, where dSpCas9–Mxi1 was produced by pGPD, showed slightly better performance when the gRNAs were synthesized via an RGR cassette under the *ADH1* promoter. However, the transcription unit pSNR52–gRNA–SUP4t was again more effective if integrated into the yeast genome rather than placed on a multicopy plasmid.

We chose three anti-CRISPR proteins for the construction of galactose-biosensing devices. LmAcrIIA2 and LmAcrIIA4 were previously shown to be efficient inhibitors of SpCas9 (and dSpCas9) in bacterial and human cells [30] and were recently tested also on *S. cerevisiae* [1]. Once bound to the gRNA, they act on SpCas9 by occluding its PAM binding site. StAcrIIA5 was proven to inhibit SpCas9 in bacterial cells [19]. However, the mechanism through which this anti-CRISPR works is unknown. According to previous reports, LmAcrIIA4 performs better than LmAcrIIA2 in human and bacterial cells. We have shown that, in *S. cerevisiae*, the performance of LmAcrIIA2 is comparable to that of LmAcrIIA4. Moreover, LmAcrIIA2 allowed the construction of working biosensors with every gRNA expression system considered here.

The growth curves of yeast cells re-engineered with anti-CRISPR-based biosensors highlighted how the best-performing devices (those where the gRNAs were produced via the RGR cassette) grew much more slowly than the circuits containing an RNA polymerase III-dependent transcription unit for gRNA synthesis. This growth slowdown, however, was not associated with any drastic reduction in cell viability. In the case of LmAcrIIA4, the growth of the cells hosting the RGR cassette was increased – although it was still not comparable to that of the control strains – by using a yeast codon-optimized version of the anti-CRISPR protein. No substantial changes in circuit performance were detected between A4-RGR and yo_A4-RGR circuits.

StAcrIIA5 was also employed in a yeast codon-optimized version. However, even though the corresponding biosensors worked properly, as evidenced by the fluorescence levels observed, more serious concerns arose based on the growth curves, viability test, and dot plots. Hence, this anti-CRISPR protein does not seem to be a reliable component of synthetic circuits in *S. cerevisiae* cells.

Finally, we built *β*-estradiol-sensing devices by fusing LmAcrIIA2 to the hormone binding domain of the human estrogen receptor. The two biosensors we constructed (one with the RGR cassette, the other with pSNR52–gRNA–SUP4t on an integrative plasmid) worked effectively and reached an approximately two-fold gain when induced with 1 *μM* (A2_HBD-RGR) and 250 *nM* (A2_HBD-pSNR52i) *β*-estradiol.

Overall, our results indicate that anti-CRISPR proteins can work efficiently in *S. cerevisiae* cells. We have shown how they can be used to construct biosensing systems responding to a single chemical. Anti-CRISPR represent a useful means to control the activity of both SpCas9 and its nuclease-deficient variants. Hence, a deep understanding of their mechanisms may lead to these proteins becoming widely-used components in future yeast synthetic regulatory networks and DNA-editing circuits.

## Methods

### Plasmid construction

All integrative and multicopy plasmids realized in this work (a complete list is provided in Additional file 1: Table S6) are based on the pRSII4XX yeast shuttle-vector collection (available at Addgene–their codes are reported below–a gift from Steven Haase) [4].

The vector targeted by the CRISPR-dSpCas9 system (pMM260) contains the transcription unit Tsynth8_pCYC1noTATA-yEGFP-CYC1t. The assembly of this plasmid is described in our previous work [35].

As a template for gRNA expression via the *RPR1* promoter and terminator we used the plasmid "pRPR_gRNA_handle_RPR1t" (Addgene-49014, a gift from Timothy Lu). We constructed, via isothermal assembly [13], two plasmids carrying this expression cassette modified with the insertion of the spacer sequence ATAAACTCATTTACTTATGT. They are the integrative vector pMM218 (backbone: pRSII404, Addgene-35438) and the multicopy vector pMM219 (backbone: pRSII424, Addgene-35466).

In order to assemble a gRNA expression cassette via the *SNR52* promoter and *SUP4* terminator we constructed, via isothermal assembly, the gRNA-spacer acceptor vector pMM465 that contains the transcription unit pSNR52-BpiI(GATC)-place_for_spacer-BpiI(GTTT)-SpCas9_repeat-SUP4t. Backbone for pMM465 was pRSII404. The spacer sequence was extended with BpiI sites compatible with those present on pMM465. Primers containing the extended spacer sequence (synthesized, annealead, and purified by COMATE, Harbin, China) were inserted into pMM465 via Golden Gate method [9]–see below for details. The resulted plasmid was named pMM524. The transcription unit pSNR52-gRNA-SUP4t from pMM524 was inserted into the multicopy shuttle vector pRSII424 via digestion with KpnI (NEB-R0142S) and SacI (NEB-R0156S), and successive ligation with T4 DNA ligase (NEB-M0202S).

The RGR sequence (HH_ribozyme-spacer-SpCas9_repeat-HDV_ribozyme) followed by the *ADH1* terminator was synthesized by GENEWIZ Inc., Suzhou (China), and then assembled, with the Gibson method, into pRSII404 together with the *ADH1* promoter. The new plasmid was named pMM554.

NLS-dSpCas9 was extracted from the plasmid “pTPGI_dCas9_VP64” (Addgene-49013, a gift from Timothy Lu) via digestion with XbaI (NEB-R0145S) and SalI (NEB-R0138S).

In order to express NLS-dSpCas9 under the *GPD* promoter we constructed the acceptor vector pMM551. pGPD and CYC1t were extended via PCR–and then assembled, via Gibson method, into pRSII406 (Addgene-35442)–to have the sequence: pGPD-ATG-XbaI-space_for_NLS-dSpCas9-SalI-GG-STOP-CYC1t, where G stands for glycine. This plasmid served as a template to build pMM559, another acceptor vector for NLS-dSpCas9, where pGAL1 replaced pGPD. NLS-dSpCas9 was inserted into both plasmids after digestion with XbaI and SalI followed by ligation with T4 DNA ligase.

pMM551 and pMM559 were used to construct pMM573 and pMM574, respectively. They are the NLS-dSpCas9 acceptor vectors that contain the Mxi1 repression domain. For instance, pMM573 includes the sequence: pGPD-ATG-XbaI-space_for_NLS-dSpCas9-SalI-GG-GS-Mxi1-STOP-CYC1t. Both pMM573 and pMM574 were assembled, with the Gibson method, into pRSII406. NLS-dSpCas9 was inserted into them via digestion with XbaI and SalI, and ligation with T4 DNA ligase.

Finally, all plasmids expressing anti-CRISPR proteins were build into the pRSII403 backbone (Addgene-35436) via isothermal assembly. LmAcrIIA2, LmAcrIIA4, yo_LmAcrIIA4, and yo_StAcrIIA4 were synthesized by GENEWIZ Inc., Suzhou, China. The hormone binding domain of the human estrogen receptor was extracted, via PCR, from the plasmid "pHCA/GAL4(1-93).ER.VP16" [20] (courtesy of Didier Picard, University of Geneva, Switzerland).

Touchdown PCR was employed to extract DNA sequences from the above cited plasmids (the sequences of all parts used in this work–promoters, coding regions, and terminators–are provided in the Additional file 1). DNA elution from agarose gel was carried out with the “AxyPrep DNA extraction kit” (Axigen–AP-GX-250). Isothermal assembly required always one hour at 50°*C*. As for the Golden Gate assembly, the insert (spacer) and the acceptor vector (pMM465) were combined in a 8:1 molar ratio and mixed with a master mix (1 *μl* BpiI 10 units /*μl*, Thermo Scientific-ER1011; 2 *μl* G buffer, Thermo Scientific; 1 *μl* T4 DNA ligase 400 units /*μl*, NEB-M0202S; 2 *μl* 10 *mM* ATP, Sigma-Aldrich-A7699) to a final 15 *μl* volume. The thermocycler program was set to: 3 cycles of 10 min at 37°*C* and other 10 min at 16°*C*. These cycles were followed by 10 min at 37°*C*, 20 min at at 65°*C*, and the final temperature was set to 4°*C*.

*E. coli* competent cells (strain DH5 *α*, Life Technology 18263-012) transformed with our plasmids (30-s heatshock at 42°*C*) have been grown overnight at 37°*C* in LB broth or plates (Bacto-tryptone 10%, Yeast extract 5%, NaCl 10%, Agar 15% for the plates) supplied with ampicillin. Plasmid extraction from bacterial cells was carried out with standard methods [16]. All plasmids built via isothermal assembly have been sequenced (Sanger method) to check the correctness of their insert sequences.

### Yeast strain construction

Our integrative plasmids were placed into the genome of the yeast *S. cerevisiae* strain CEN.PK2-1C (MATa; his3 *Δ*1; leu2-3_112; ura3-52; trp1-289; MAL2-8c; SUC2), Euroscarf (Johann Wolfgang Goethe University, Frankfurt, Germany). Genomic integration was carried out as described in [14]. About 5 *μg* of plasmidic DNA were linearized at the corresponding auxotrophic marker with a proper restriction enzyme. Transformed cells were grown on plates containing synthetic selective medium (2% glucose, 2% agar) from 2 up to 4 days at 30°*C*. A similar procedure (without plasmid linearization) was followed to transform yeast cells with multicopy plasmids. A list of all synthetic yeast strains realized in this work is given in Additional file 1: Table S7.

### Flow cytometry

Yeast cells were grown overnight in synthetic complete medium (SDC containing either 2% glucose or 2% galactose) at 30°*C*. They were diluted, in the morning, approximately 1:100 and let grow in SDC up to five more hours such that their *OD600* was always between 0.2 and 2.0 (exponential phase). When *β*-estradiol (Sigma-Aldrich–E8875) was used to induce the translocation of the anti-CRISPR proteins into the nucleus, yeast cells were grown overnight in synthetic complete medium (SDC with 2% glucose) at 30°*C*. They were diluted, in the morning, approximately 1:100 and let grow in SDC up to five more hours. Cells were then diluted into SDC supplied with *β*-estradiol (concentrations up to 8 *μM*) and grown overnight. In the morning, cells were diluted, again, roughly 1:100 in SDC plus *β*-estradiol. FACS experiments were run after four-five hours upon reaching the exponential phase.

Fluorescence measurements were performed with a BD FACScalibur machine (488nm laser, 530/30 filter). The FACS machine set-up was reproduced at each experiment by using fluorescent beads (AlignFlown Life Technologies-A16500). We placed their peak (mean value) as close as possible to 400 AU. The measurement was repeated at the end of each experiment to assure that the machine conditions did not change considerably over the whole experiment. We considered as reliable only the measurements where the relative difference between the initial and the final value of the peaks of the beads was lower than 5%. Data were analyzed with the flowcore R-Bioconductor package [17]. As referenced in the main text, statistical analysis was conducted via two-sided Welch’s t-test (p-value < 0.05). Fluorescence levels were estimated as the mean values of at least three independent experiments (i.e. carried out in different days–each time 30000 samples were recorded). Standard deviations were calculated on these mean values.

### RT-qPCR

Purified RNA was extracted from yeast cells with the YeaStar RNA kit (Zymo Research-R1002). In order to synthesize cDNA, an RNA-oligo solution was first prepared by mixing 2 *μg* of purified RNA together with 2 *μl* of 9-nt-long random primers (Takara-3802), and nuclease-free water up to an overall volume of 5 *μl*. The RNA-oligo solution was heated at 70°*C* for 5 min, then kept on ice for 5 more min, and finally centrifuged for 10 s. At this point, the whole 5 *μl* of RNA-oligo solution were added, into a PCR tube, to a 15 *μl* “RT mix” (1 *μl* GoScript Reverse Transcriptase–Promega, A5003; 4 *μl* GoScript 5*X* RT buffer; 1.5 *μl* *MgCl*_2_; 4 *μl* 0.5 *mM* dNTPmix; nuclease-free water up to the overall volume of 15 *μl*). The PCR tube containing the RNA-oligo solution together with the RT mix was placed in a thermal shaker and the following program was run: 5 min at 25°*C*; 1 h at 42°*C*; 15 min at 70°*C*. The obtained cDNA was used as a template for qPCR. The guide RNA targeting the Tsynth8_pCYC1noTATA synthetic promoter was amplified with forward primer (oMM940) 5’-TAAACTCATTTACTTATGTGTTTTAGAG-3’ and reverse primer (oMM941) 5’-GACTCGGTGCCACTTTTT-3’. *ACT1* was chosen as reference gene and amplified with forward primer (oMM919) 5’-CAGGTATTGCCGAAAGAA-3’ and reverse primer (oMM920) 5’-CCACATTTGTTGGAAGGTA-3’. A variable quantity of cDNA (up to 50 *ng*) was mixed with: 5 *μl* SYBR premix (Takara-RR820A); 0.5 *μl* of 10 *μM* forward and reverse primers; 0.2 *μl* ROX(II), and water up to an overall volume of 10 *μl*. qPCR was run on an Applied Biosystems ViiA 7 machine with the following protocol: 1) Hold stage: 2 min at 50°*C* followed by 10 min at 95°*C*; 2) PCR stage: 15 s at 95°*C* followed by 34 s at 58°*C*. The PCR stage was cycled 45 times. qPCR permitted the estimation of the threshold cycle for ACT1 gene and the guide RNA. Each sample was present in three replicates.

We estimated the efficiency of amplification of both pairs of primers used in our experiments by calculating their standard curve (see Additional file 1: Figure S11). The ratio between the amount of gRNA and mRNA corresponding to the *ACT1* gene was calculated with the Pfaffl formula [29].

### Growth curves

Yeast cells were grown overnight (either in SDC or SD-TRP supplied with 2% glucose or 2% galactose) at 30°*C*. They were diluted, in the morning, approximately 1:100 and grown in the corresponding medium up to six more hours. Cells were then diluted to roughly *OD600* =0.2 into 2 ml of the corresponding growth medium and poured into 24-well plates–in triplicates. Finally, yeast cells were grown for 18 h inside a TECAN NanoQuant Infinite M200 machine (orbital shaking: amplitude 1 mm, frequency: 87.5 RPM). *OD600* was measured every 10 min.

### Viability test

A 0.08*%* solution of trypan blue (Beyotime–ST798) was prepared by dissolving trypan blue powder in water. Yeast cells were grown overnight in synthetic medium supplied with either 2% galactose or 2% glucose (with 1 *μM* *β*-estradiol for the two hormone-sensing circuits) then diluted to *OD600* between 0.2 and 0.4. Yeast cells were then stained with trypan blue (1:1 ratio, 200 *μl* for each solution) and, after 5÷10 min, loaded on a hemocytomer (MC Qiujing–02270113). The viability coefficient, defined as the number of alive cells divided by the total number of cells, was evaluated into 4 quadrants of the hemocytometer. Mean value and corresponding standard deviation were finally calculated.

## Additional file

Additional file 1 Supplementary Material. (PDF 1621 kb)

## Abbreviations

ADH1t: *ADH1* terminator
CRISPR: Clustered regularly interspaced short palindromic repeats
CYC1t: *CYC1* terminator
dSpCas9: Nuclease-deficient SpCas9
FACS: Fluorescence activated cell sorting
gRNA: Guide RNA
HBD(ER): Hormone binding domain of the human estrogen receptor
HDV: Hepatitis delta virus (ribozyme)
HH: Hammerhead (ribozyme)
Hsp90: Heat shock protein 90
LB: Luria-Bertani (medium)
LmAcrIIA2/A4: *L. monocytogenes* anti-CRISPR type II-A number 2/4 (protein)–type IIA refers to the inhibited CRISPR-Cas system
NLS: Nuclear localization sequence
pADH1: *ADH1* promoter
PAM: protospacer adjacent motif
pCYC1noTATA: Minimal *CYC1* promoter without TATA boxes
pGAL1: *GAL1* promoter
pGPD: *GPD* promoter
pRPR1: *RPR1* promoter
pRPR1i: pRPR1-gRNA-RPR1t on integrative plasmids
pRPR1m: pRPR1-gRNA-RPR1t on multicopy plasmids
pSNR52: *SNR52* promoter
pSNR52i: pSNR52-gRNA-SUP4t on integrative plasmids
pSNR52m: pSNR52-gRNA-SUP4t on multicopy plasmids
RGR: ribozyme-gRNA-ribozyme
RPR1t: *RPR1* terminator
RT-qPCR: Reverse transcription quantitative polymerase chain reaction
SDC: Synthetic complete medium
SpCas9: *S. pyogenes* CRISPR-associated 9 (protein)
StAcrIIA5: *S. thermophilus* anti-CRISPR type II-A number 5 (protein)
SUP4t: *SUP4* terminator
yEGFP: Yeast enhanced green fluorescent protein
yo_LmAcrIIA4/A5: yeast codon-optimized LmAcrIIA4/A5
