# Supplementary material for: Anti-CRISPR-based biosensors in the yeast S. cerevisiae
Source: J Biol Eng. 2018 Aug 1;12:11. doi: 10.1186/s13036-018-0101-z (PMC6090965; doi:10.1186/s13036-018-0101-z)
Supplement: Supplementary file 1 — Supplementary Material. (PDF 1621 kb) [file 13036_2018_101_MOESM1_ESM.pdf]

Anti-CRISPR–based biosensors in the yeast *S. cerevisiae*
Supplementary Material

Jing Li[1§], Zengliang Xu[1§], Aleksandr Chupalov[1§], and Mario Andrea Marchisio[1,*]

[1]School of Life Science and Technology, Harbin Institute of Technology,

2 Yikuang Street, Nan Gang District, 150080 Harbin, P. R. China

[§] These authors contributed equally to this work

[*]corresponding author

email addresses: JL, 920138462@qq.com; ZX, 2734001081@qq.com; AC, biochup@yandex.ru

MAM: marchisio@hit.edu.cn, mamarchisio@yahoo.com

Table S1: Relative fluorescence of the eight NOT gates built in this work. Numerical values of the average OFF/ON ratio–with the corresponding standard deviation, σ_M–associated with the eight NOT gates (see main text Figure 1B) are here given. For each gate, the p-value is calculated via two-sided Welch's t-test to evaluate if a statistically significant difference exists between the fluorescence levels measured in the presence of glucose (ON state) and galactose (OFF state). NOT gates were evaluated in a variable number of independent experiments (replicates). Since every p-value < 0.05, every NOT gate appears to discriminate between ON and OFF states properly.

NOT gate	Mean OFF/ON ratio	σ_M	p-value	Replicates
RGR	0.31	0.03	0.006	3
RGR-Mxi1	0.282	0.005	0.024	3
pSNR52i	0.36	0.06	0.011	4
pSNR52i-Mxi1	0.17	0.03	0.005	4
pSNR52m	0.30	0.06	$2.9\,10^{-5}$	4
pSNR52m-Mxi1	0.36	0.04	$6.2\,10^{-4}$	4
pRPR1i-Mxi1	0.33	0.08	$2.1\,10^{-4}$	6
pRPR1m-Mxi1	0.37	0.07	$1.1\,10^{-4}$	5

Table S2: Relative fluorescence of the three-gene sub-circuits used for the construction of the anti-CRISPR-based biosensors. For each circuit, the p-value is calculated via two-sided Welch's t-test to evaluate if a statistically significant difference exists between the mean fluorescence measured in the presence and absence of the dSpCas9-Mxi1:gRNA system. σ_M indicates the standard deviation on each average relative fluorescence level. Every circuit was evaluated in 3 independent experiments.

Circuit	Relative fluorescence	σ_M	p-value
RGR-Mxi1	0.13	0.01	0.0103
pSNR52i-Mxi1	0.25	0.03	0.0007
pSNR52m-Mxi1	0.36	0.03	0.0202

NOT gates	**RGR**	**RGR-Mxi1**	**pSNR52i**	**pSNR52i-Mxi1**	**pSNR52m**	**pSNR52m-Mxi1**	**pRPR1i-Mxi1**	**pRPR1m-Mxi1**
**RGR**	-	0.445	0.233	**0.004**	0.984	0.089	0.544	0.141
**RGR-Mxi1**	0.445	-	0.139	**0.007**	0.682	**0.042**	0.030	0.069
**pSNR52i**	0.233	0.139	-	**0.007**	0.300	0.913	0.585	0.906
**pSNR52i-Mxi1**	**0.004**	**0.007**	**0.007**	-	**0.022**	**0.001**	**0.006**	**0.001**
**pSNR52m**	0.984	0.682	0.300	**0.022**	-	0.194	0.608	0.225
**pSNR52m-Mxi1**	0.089	**0.042**	0.913	**0.001**	0.194	-	0.454	0.981
**pRPR1i-Mxi1**	0.544	0.030	0.585	**0.006**	0.608	0.454	-	0.487
**pRPR1m-Mxi1**	0.141	0.069	0.906	**0.001**	0.225	0.981	0.487	-

Figure S1: NOT gate comparison via two-sided Welch's t-test. The eight NOT gates we constructed are analyzed in pairs via two-sided Welch's t-test (p-values are the matrix elements). Statistically significant difference is highlighted in green. pSNR52i-Mxi1 shows statistically significant difference with respect to any other configuration. Moreover, RGR-Mxi1 and pSNR52m-Mxi1 are statistically different in a significant way too.

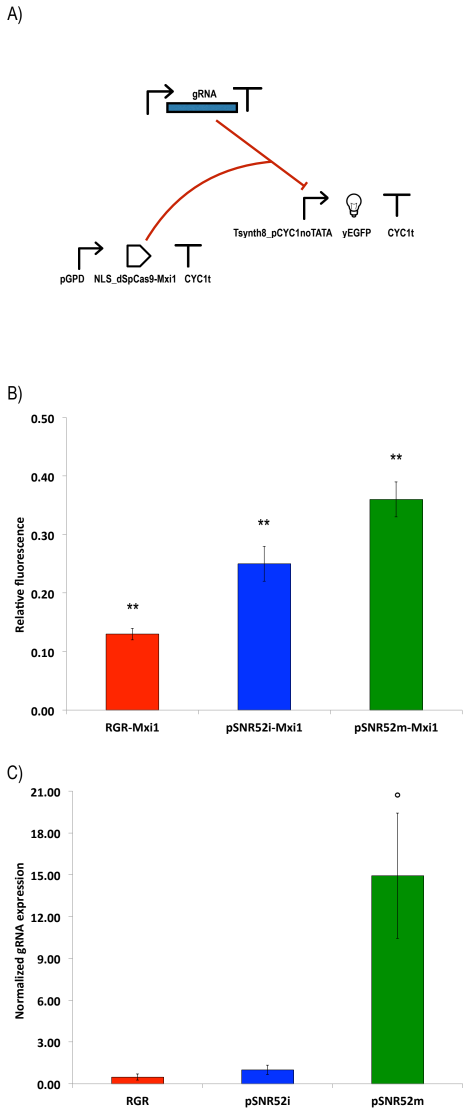

Figure S2: Three-gene circuits for the construction of anti-CRISPR-based biosensors. The Figure caption is reported below.

Figure S2 caption. Three-gene circuits for the construction of anti-CRISPR-based biosensors. A) Circuit scheme. The guide RNA is expressed either via an RGR cassette or the RNA polymerase III-dependent *SNR52* promoter. dSpCas9-Mxi1 is constitutively expressed under *GPD* promoter. B) Relative fluorescence between the output signals in presence and absence of the dSpCas9-Mxi1:gRNA complex. Each circuit is labelled with the abbreviation for the transcription unit leading the gRNA expression (as in main text Figure 1B) followed by "Mxi1". The highest fluorescence repression was achieved by expressing the guide RNA through the RGR cassette (RGR-Mxi1). The configuration where pSNR52 is placed on an integrative plasmids (pSNR52i-Mxi1) turned out to be more efficient than that where the gRNA is expressed via a multicopy plasmid (pSNR52m-Mxi1). The three mean relative fluorescence values (each obtained from 3 independent experiments) are significantly different from each other in statistical terms (as indicated by the "$**$" symbol–two-sided Welch's t-test, p-value < 0.05.) C) Normalized gRNA expression. For each circuit, the relative amount of gRNA with respect to the mRNA of the *ACT1* gene was first calculated (mean value from three replicates during a single experiment–$12.5\,ng$ of cDNA were used). Then, gRNA relative expressions were normalized to the value obtained for pSNR52i-Mxi1, in analogy with the measurements on the NOT gates (see main text Figure 1C). Error bars were calculated on the normalized values by means of the error propagation formula. The *ADH1* promoter present in the RGR-Mxi1 configuration produces an amount of gRNA (0.48 ± 0.22) comparable to that of pSNR52 on an integrative plasmid (pSNR52i-Mxi1: 1.00 ± 0.34) since a comparison via the two-sided Welch's t-test returned a p-value equal to $= 0.076$. In contrast, pSNR52m-Mxi1–pSNR52 is inside a multicopy plasmid–expresses 14.93 (± 4.50)-fold more gRNA than pSNR52i-Mxi1 (the "$\circ$" symbol points out a statistically significant difference with respect to the reference gate–two-sided Welch's t-test, p-value < 0.05). These results are in agreement with those from the NOT gates and confirm that gRNA expression does not play a major role in determining the fluorescence repression observed in these circuits.

Table S3: Relative fluorescence of the ten YES gates (sensing galactose) built in this work. Numerical values of the average ON/OFF ratio–with the corresponding standard deviation, σ_M–associated with the ten YES gates (see main text Figure 2B) are here given. For each gate, the p-value is calculated via two-sided Welch's t-test to evaluate if a statistically significant difference exists between the fluorescence levels measured in the presence of galactose (ON state) and glucose (OFF state). YES gates were evaluated in a variable number of independent experiments (replicates). Only two gates (in italics) are not working since their p-value ≥ 0.05–they do not discriminate between the ON and the OFF states properly.

YES gate	Mean ON/OFF ratio	σ_M	p-value	Replicates
A4-RGR	3.16	0.49	0.027	4
yo_A4-RGR	2.87	0.57	0.001	5
A4-pSNR52i	1.30	0.14	0.180	4
yo_A4-pSNR52i	1.41	0.27	0.047	5
A4-pSNR52m	0.92	0.26	0.680	5
A2-RGR	3.94	0.52	0.005	4
A2-pSNR52i	3.17	1.11	0.024	6
A2-pSNR52m	2.41	0.59	0.029	3
yo_A5-RGR	1.86	0.76	0.018	8
yo_A5-pSNR52i	1.76	0.45	0.015	6

YES gates	**A4-RGR**	**yo_A4-RGR**	**A4-pSNR52i**	**yo_A4-pSNR52i**	**A4-pSNR52m**	**A2-RGR**	**A2-pSNR52i**	**A2-pSNR52m**	**yo_A5-RGR**	**yo_A5-pSNR52i**
**A4-RGR**	-	0.496	**0.005**	**0.004**	**0.002**	0.108	0.989	0.216	**0.011**	**0.007**
**yo_A4-RGR**	0.496	-	**0.005**	**0.005**	**0.001**	**0.037**	0.690	0.418	**0.031**	**0.015**
**A4-pSNR52i**	**0.005**	**0.005**	-	0.633	**0.031**	**0.002**	0.063	0.118	0.117	0.114
**yo_A4-pSNR52i**	**0.004**	**0.005**	0.633	-	**0.030**	**0.001**	0.068	0.127	0.193	0.215
**A4-pSNR52m**	**0.002**	**0.001**	**0.031**	**0.030**	-	**0.0007**	**0.04**	0.059	**0.015**	**0.011**
**A2-RGR**	0.108	**0.037**	**0.002**	**0.001**	**0.0007**	-	0.334	**0.042**	**0.001**	**0.001**
**A2-pSNR52i**	0.989	0.690	0.063	0.068	**0.04**	0.334	-	0.368	0.131	0.110
**A2-pSNR52m**	0.216	0.418	0.118	0.127	0.059	**0.042**	0.368	-	0.333	0.252
**yo_A5-RGR**	**0.011**	**0.031**	0.117	0.193	**0.015**	**0.001**	0.131	0.333	-	0.782
**yo_A5-pSNR52i**	**0.007**	**0.015**	0.114	0.215	**0.011**	**0.001**	0.110	0.252	0.782	-

Figure S3: YES gate comparison via two-sided Welch's t-test. Ten YES gates that respond to galactose (in red the name of the two non-working configurations) are analyzed in pairs via two-sided Welch's t-test (p-values are the matrix elements). Statistically significant difference is highlighted in green.

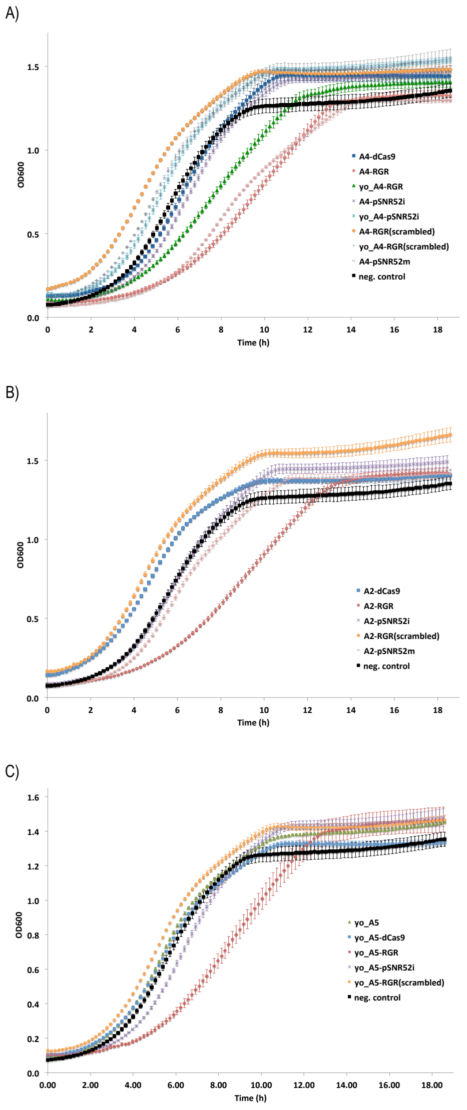

Figure S4: Growth curves in synthetic medium supplied with 2% glucose. A) LmAcrIIA4- and yo_LmAcrIIA4-based biosensors. B) LmAcrIIA2-containing biosensors. C) yo_StAcrIIA5-based biosensors. Every OD600 mean value was calculated on three replicas (single experiment).

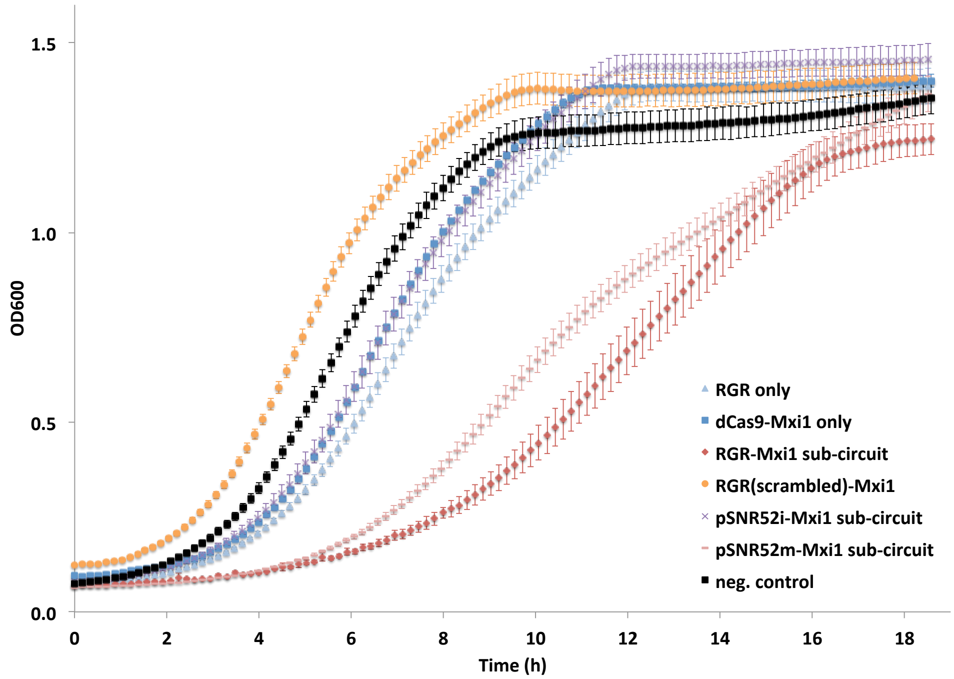

Figure S5: Growth curves of the sub-circuits used in the construction of the anti-CRISPR-based biosensors. The yeast strain byMM234 ("neg. control"), which expresses yEGFP under the synthetic promoter Tsynth8_pCYC1noTATA, shows similar growth rates when modified with the insertion of a single transcription unit for the expression of either the gRNA (via the RGR cassette–"RGR only") or the dSpCas9-Mxi1 protein ("dCas9-Mxi1 only"). Interestingly, the three-gene pSNR52i-Mxi1 sub-circuit–where the gRNA is expressed under the RNA polymerase III-type *SNR52* promoter on an integrative plasmid–presents a growth curve almost overlapping that associated with dCas9-Mxi1. Cells hosting the other two three-gene sub-circuits (RGR-Mxi1 and pSNR52m-Mxi1) grow more slowly. When the RGR expresses a "scrambled" gRNA unable to bind anywhere along the *S. cerevisiae* genome, a fast growth curve is achieved again.

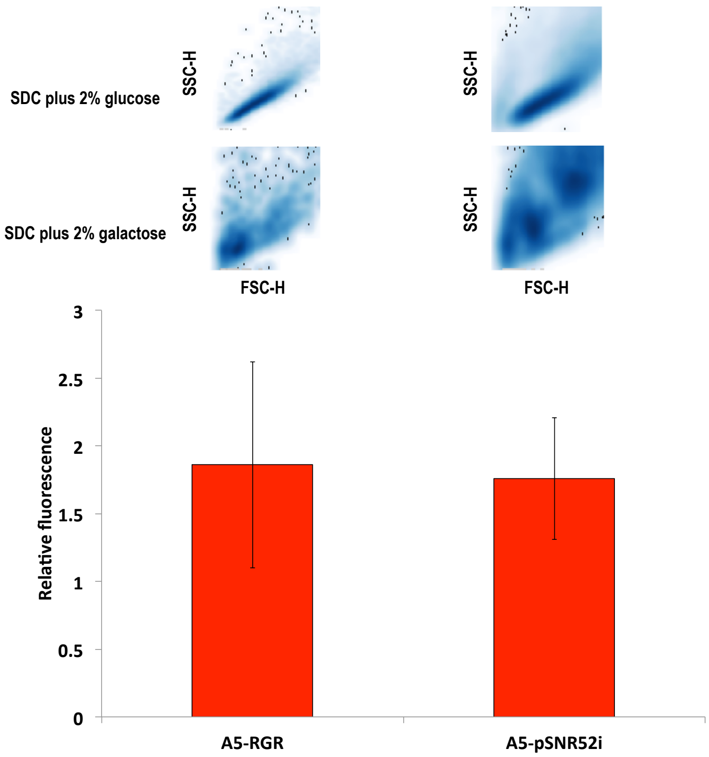

Figure S6: Dot plots from FACS experiments on yo_StAcrIIA5-based biosensors. Both biosensors hosting yo_StAcrIIA5 show a regular yeast cell population shape only after growing in synthetic medium supplied with glucose. In the presence of galactose a higher variability in cell dimension appears, especially in the yo_A5-pSNR52i circuit.

Table S4: Viability coefficients (galactose biosensors). The mean values of the viability coefficients (see main text Figure 2C) together with their standard deviation, σ_M, were calculated over four replicates for each circuit (single experiment).

YES gate	Viab. coeff.	σ_M
A4-RGR	0.84	0.02
yo_A4-RGR	0.91	0.03
A2-RGR	0.83	0.04
yo_A5-RGR	0.71	0.02
yo_A5-pSNR52i	0.80	0.09
control	0.985	0.004

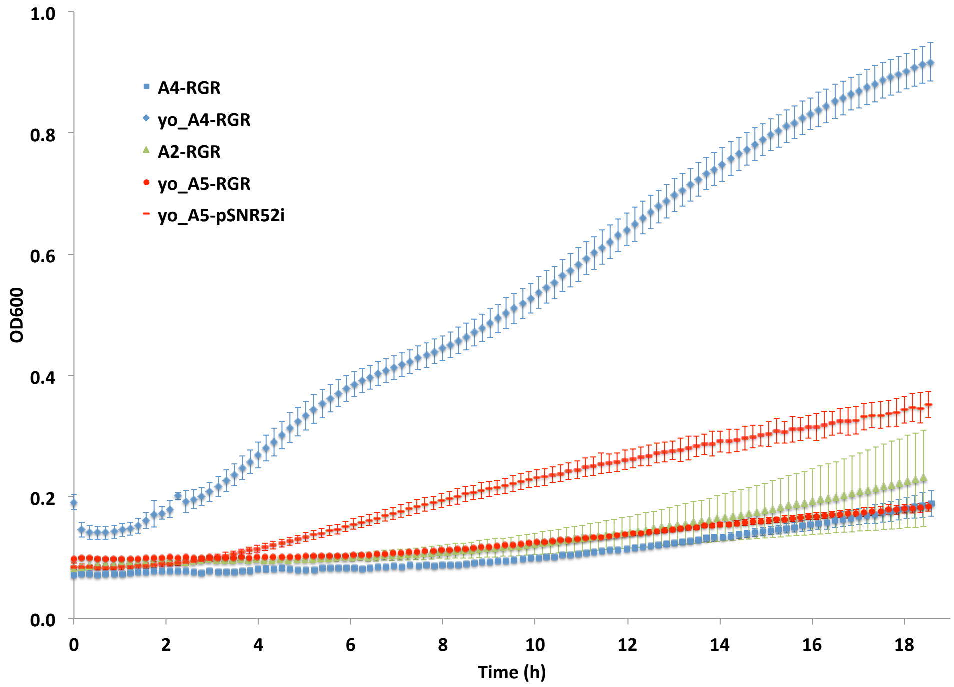

Figure S7: Comparison of the growth curves of the YES gates whose viability coefficients are given in Table S4.

YES gates	A4-RGR	yo_A4-RGR	A2-RGR	yo_A5-RGR	yo_A5-pSNR52i	control
**A4-RGR**	-	**0.024**	0.614	**0.0001**	0.464	**0.0002**
**yo_A4-RGR**	**0.024**	-	**0.029**	**0.0002**	0.114	**0.025**
**A2-RGR**	0.614	**0.029**	-	**0.006**	0.613	**0.005**
**yo_A5-RGR**	**0.0001**	**0.0002**	**0.006**	-	0.181	**8.3E-05**
**yo_A5-pSNR52i**	0.464	0.114	0.613	0.181	-	**0.036**
**control**	**0.0002**	**0.025**	**0.005**	**8.3E-05**	**0.036**	-

Figure S8: Statistical comparison of the viability coefficients of five YES gates. The two-sided Welch's t-test was used to compare the viability coefficient of the five YES gates in Table S4 plus their negative control; p-values are the matrix elements. Statistically significant difference is highlighted in green.

A)

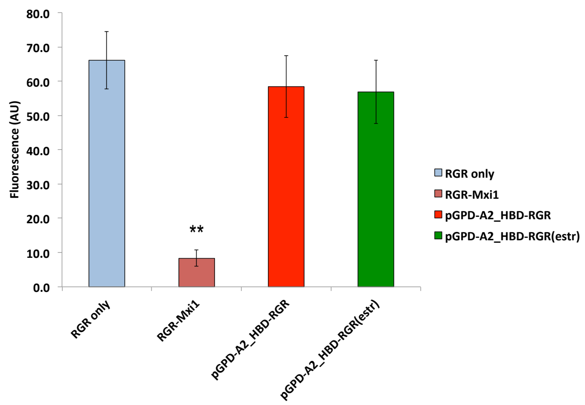

B)

circuits	RGR only	RGR-Mxi1	pGPD-A2_HBD-RGR	pGPD-A2_HBD-RGR(estr)
**RGR only**	-	**0.010**	0.324	0.259
**RGR-Mxi1**	**0.010**	-	**6.624E-07**	**1.855E-06**
**pGPD-A2_HBD-RGR**	0.324	**6.624E-07**	-	0.122
**pGPD-A2_HBD-RGR(estr)**	0.259	**1.855E-06**	0.122	-

Figure S9: Variant of the β-estradiol biosensor where the chimeric protein LmAcrIIA2-HBD(ER) is expressed under the strong constitutive *GPD* promoter. The Figure caption is reported below.

Figure S9 caption. Variant of the β-estradiol biosensor where the chimeric protein LmAcrIIA2-HBD(ER) is expressed under the strong constitutive *GPD* promoter. A) Fluorescence levels. "RGR only" is a two-gene circuit where, together with yEGFP, the sole gRNA is expressed (high fluorescence level). RGR-Mxi1 is the three-gene sub-circuit on which we built one β-estradiol biosensor. The fluorescence expressed by RGR-Mxi1 is low due to the action of dSpCas9-Mxi1:gRNA on the target promoter Tsynth8_pCYC1noTATA. pGPD-A2_HBD-RGR is the four-gene circuit where LmAcrIIA2-HBD(ER) is synthesized under *GPD* promoter. In absence of β-estradiol (red bar), the fluorescence level should be comparable with that of RGR-Mxi1. However, we detected a much higher signal, similar to that of "RGR only". This can be explained with the fact that the strong *GPD* promoter leads to the production of a large quantity of LmAcrIIA2-HBD(ER) proteins. Hence, some of them are not affected by the interactions with Hsp90 and enter the cell nucleus, where they bind and inhibit the dSpCas9-Mxi1:gRNA system, even in the absence of β-estradiol. The green bar (pGPD-A2_HBD-RGR(estr)) gives the fluorescence level of the circuit pGPD-A2_HBD-RGR upon induction with $1000\,nM$ β-estradiol. B) Statistical comparison of the four fluorescence levels in (A) via two-sided Welch's t-test. Only the fluorescence expressed by RGR-Mxi1 is statistically different in a significant way from the other three ("**" symbol in (A); p-values < 0.05 are highlighted in green). "RGR", pGPD-A2_HBD-RGR, and pGPD-A2_HBD-RGR(estr) are, in contrast, statistically undistinguishable. Hence, pGPD cannot be used to build β-estradiol-sensing circuits.

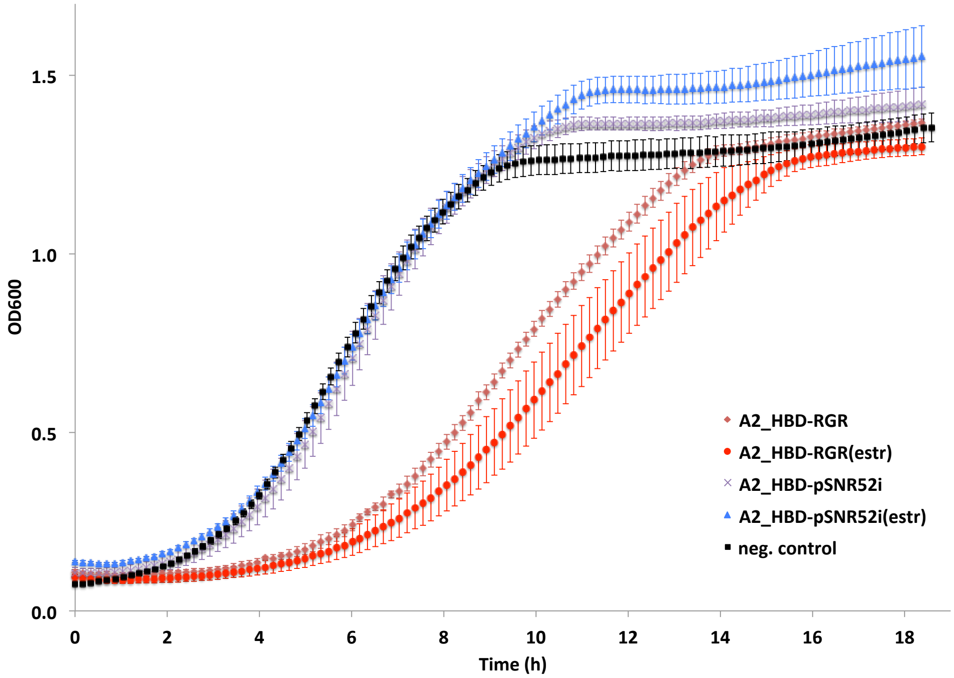

Figure S10: Growth curves of β-estradiol-sensing circuits. As in the case of the galactose biosensors, the RGR cassette appears to slow down circuit growth rate, both in the presence and absence of β-estradiol. However, this effect is not as dramatic as in the A2-RGR circuit (see main text Figure 3B). The gRNA expression system based on pSNR52 on an integrative plasmid has no apparent negative effects on cell growth. Both A2_HDB-RGR(estr) and A2_HBD-pSNR52i(estr) were induced with $1\,\mu M$ β-estradiol, enough to assure a maximal fluorescence gain (see main text Figure 4B-C). Each value of OD600 was measured in three replicates (only two for A2_HDB-RGR) during a single experiment.

Table S5: Viability coefficients (β-estradiol biosensors). The mean values of the viability coefficients, together with their standard deviation σ_M, were calculated over four replicates for each circuit (single experiment).

YES gate	Viab. coeff.	σ_M
A2_HDB-RGR	0.994	0.002
A2_HDB-RGR(estr)	0.998	0.004
A2_HDB-pSNR52i	0.997	0.001
A2_HDB-pSNR52i(estr)	0.998	0.001

Plasmids realized in this work

Table S6: List of the plasmids assembled in this work.

Plasmid name	Construct
pMM218	pRSII404-pRPR1-gRNA-RPR1t
pMM219	pRSII424-pRPR1-gRNA-RPR1t
pMM260	pRSII405-Tsynth8_pCYC1noTATA-yEGFP-CYC1t
pMM465	pRSII404-pSNR52-BpiI(GATC)-place_for_spacer-BpiI(GTTT)-SpCas9_repeat-SUP4t
pMM522	pRSII403-pGAL1-LmAcrIIA4-CYC1t
pMM524	pRSII404-pSNR52-gRNA-SUP4t
pMM536	pRSII424-pSNR52-gRNA-SUP4t
pMM551	pRSII406-pGPD-ATG-XbaI-space_for_NLS-dSpCas9-SalI-GGTGGA-STOP-CYC1t
pMM554	pRSII404-pADH1-RGR-ADH1t
pMM559	pRSII406-pGAL1-ATG-XbaI-space_for_NLS-dSpCas9-SalI-GGTGGA-STOP-CYC1t
pMM562	pRSII406-pGPD-NLS-dSpCas9-CYC1t
pMM563	pRSII406-pGAL1-NLS-dSpCas9-CYC1t
pMM573	pRSII406-pGPD-ATG-XbaI-space_for_NLS-dSpCas9-SalI-GGTGGA-GS-Mxi1-STOP-CYC1t
pMM574	pRSII406-pGAL1-ATG-XbaI-spacer-SalI-GGTGGA-GS-Mxi1-STOP-CYC1t
pMM578	pRSII406-pGPD-dSpCas9-GS-Mxi1-CYC1t
pMM579	pRSII406-pGAL1-dSpCas9-GS-Mxi1-CYC1t
pMM609	pRSII403-pGAL1-LmAcrIIA2-CYC1t
pMM625	pRSII403-pGAL1-yo_StAcrIIA5-CYC1t
pMM626	pRSII403-pGAL1-yo_LmAcrIIA4-CYC1t
pMM637	pRSII403-pTEF2-LmAcrIIA2-GS-HBD(ER)-CYC1t
pMM676	pRSII403-pGPD-LmAcrIIA2-GS-HBD(ER)-CYC1t
pMM685	pRSII404-pADH1-RGR(scrambled gRNA)-ADH1t

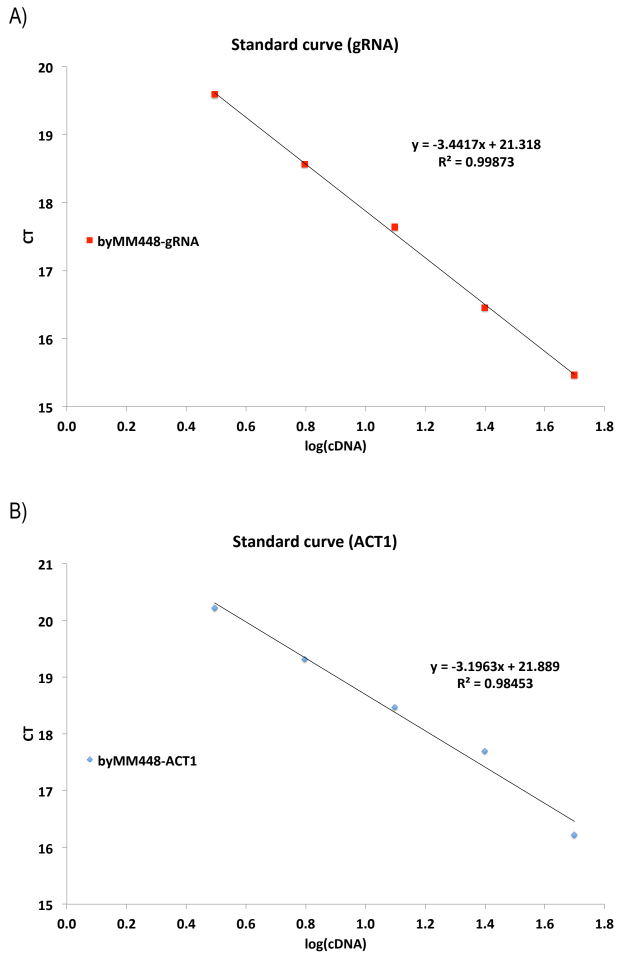

Figure S11: Standard curves. cDNA (diluted from 50 ng to 3.125 ng) from the strain byMM448 was used as a template for both standard curves. A) A portion (90 nt) of the gRNA targeting the synthetic promoter Tsynth8_pCYC1noTATA was amplified with primers oMM940/941. The amplification efficiency resulted equal to 1.95. B) Primers oMM919/920 were used to PCR out a trait of the $ACT1$ gene (149 nt). In this case, the amplification efficiency turned out to be 2.06.

Yeast strains used/realized in this work

Table S7: List of yeast strains, realized in this work (plus the original one, byMM111). Plasmids are described in Tables S6. ∗: transformed with the multicopy plasmid pMM536; ∗∗: transformed with the multicopy plasmid pMM219.

Strain name	Genotype
byMM111	CEN.PK2-1C (MATa; his3D1; leu2-3_112; ura3-52; trp1-289; MAL2-8c; SUC2)
byMM234	byMM111 pMM260::LEU2
byMM376	byMM111 pMM260::LEU2 pMM554::TRP1
byMM387	byMM111 pMM260::LEU2 pMM554::TRP1 pMM563::URA3
byMM389	byMM111 pMM260::LEU2 pMM554::TRP1 pMM579::URA3
byMM395	byMM111 pMM260::LEU2 pMM554::TRP1 pMM578::URA3
byMM398	byMM111 pMM260::LEU2 pMM579::URA3
byMM399	byMM111 pMM260::LEU2 pMM578::URA3
byMM400	byMM111 pMM260::LEU2 pMM579::URA3
byMM407	byMM111 pMM260::LEU2 pMM554::TRP1 pMM578::URA3 pMM522::HIS3
byMM408	byMM111 pMM260::LEU2 pMM554::TRP1 pMM578::URA3 pMM522::HIS3
byMM410	byMM111 pMM260::LEU2 pMM578::URA3 pMM522::HIS3
byMM418	byMM111 pMM260::LEU2 pMM554::TRP1 pMM578::URA3 pMM609::HIS3
byMM420	byMM111 pMM260::LEU2 pMM578::URA3 pMM522::HIS3 *
byMM423	byMM111 pMM260::LEU2 pMM524::TRP1 pMM578::URA3
byMM425	byMM111 pMM260::LEU2 pMM554::TRP1 pMM578::URA3 pMM626::HIS3
byMM426	byMM111 pMM260::LEU2 pMM554::TRP1 pMM578::URA3 pMM625::HIS3
byMM427	byMM111 pMM260::LEU2 pMM524::TRP1 pMM578::URA3 pMM522::HIS3
byMM428	byMM111 pMM260::LEU2 pMM524::TRP1 pMM578::URA3 pMM626::HIS3
byMM429	byMM111 pMM260::LEU2 pMM524::TRP1 pMM578::URA3 pMM609::HIS3
byMM430	byMM111 pMM260::LEU2 pMM524::TRP1 pMM578::URA3 pMM625::HIS3
byMM446	byMM111 pMM260::LEU2 pMM524::TRP1 pMM579::URA3
byMM448	byMM111 pMM260::LEU2 pMM579::URA3 *
byMM450	byMM111 pMM260::LEU2 pMM578::URA3 *
byMM458	byMM111 pMM260::LEU2 pMM554::TRP1 pMM578::URA3 pMM637::HIS3
byMM459	byMM111 pMM260::LEU2 pMM524::TRP1 pMM578::URA3 pMM637::HIS3
byMM460	byMM111 pMM260::LEU2 pMM579::URA3 **
byMM461	byMM111 pMM260::LEU2 pMM218::TRP1 pMM579::URA3
byMM463	byMM111 pMM260::LEU2 pMM578::URA3 pMM609::HIS3 *
byMM469	byMM111 pMM260::LEU2 pMM524::TRP1 pMM563::URA3
byMM470	byMM111 pMM260::LEU2 pMM563::URA3 *
byMM505	byMM111 pMM260::LEU2 pMM578::URA3 pMM609::HIS3
byMM506	byMM111 pMM260::LEU2 pMM578::URA3 pMM625::HIS3
byMM507	byMM111 pMM260::LEU2 pMM625::HIS3
byMM514	byMM111 pMM260::LEU2 pMM685::TRP1 pMM578::URA3
byMM522	byMM111 pMM260::LEU2 pMM685::TRP1 pMM578::URA3 pMM609::HIS3
byMM523	byMM111 pMM260::LEU2 pMM685::TRP1 pMM578::URA3 pMM522::HIS3
byMM524	byMM111 pMM260::LEU2 pMM685::TRP1 pMM578::URA3 pMM626::HIS3
byMM525	byMM111 pMM260::LEU2 pMM685::TRP1 pMM578::URA3 pMM625::HIS3
byMM528	byMM111 pMM260::LEU2 pMM554::TRP1 pMM578::URA3 pMM676::HIS3

DNA part sequences

Promoters

pSNR52

CTTTGAAAAGATAATGTATGATTATGCTTTCACTCATATTTATACAGAAACTTGATG
TTTTCTTTCGAGTATATACAAGGTGATTACATGTACGTTTGAAGTACAACTCTAGAT
TTTGTAGTGCCCTCTTGGGCTAGCGGTAAAGGTGCGCATTTTTTCACACCCTACAAT
GTTCTGTTCAAAAGATTTTGGTCAAACGCTGTAGAAGTGAAAGTTGGTGCGCATGT
TTCGGCGTTCGAAACTTCTCCGCAGTGAAAGATAAATGATC

pRPR1

GGGGGATCTGCCAATTGAACATAACATGGTAGTTACATATACTAGTAATATGGTTCG
GCACACATTAAAAGTATAAAAACTATCTGAATTACGAATTACATATATTGGTCATAA
AAATCAATCAATCATCGTGTGTTTTATATGTCTCTTATCTAAGTATAAGAATATCCAT
AGTTAATATTCACTTACGCTACCTTTTAACCTGTAATCATTGTCAACAGGATATGTTA
ACGACCCACATTGATAAACGCTAGTATTTCTTTTTCCTCTTCTTATTGGCCGGCTGTC
TCTATACTCCCCTATAGTCTGTTTCTTTTCGTTTCGATTGTTTTACGTTTGAGGCCTC
GTGGCGCACATGGTACGCTGTGGTGCTCGCGGCTGGGAACGAAACTCTGGGAGCTGC
GATTGGCAGAAGCTT

Tsynth8_pCYC1noTATA

TATATAAACTCATTTACTTATGTAGGAATAAAGAGTATCATCTTTCAAATTCTTTCCTT
ATACATtAGGACCTTTGCAGCATAAATTACTATACTTCTATAGACACACAAACACAAATA
CACACACTAAATTAATA

pGAL1

ATATACATATCCATATCTAATCTTACTTATATGTTGTGGAAATGTAAAGAGCCCCATTAT
CTTAGCCTAAAAAAACCTTCTCTTTGGAACTTTCAGTAATACGCTTAACTGCTCATTGC
TATATTGAAGTACGGATTAGAAGCCGCCGAGCGGGTGACAGCCCTCCGAAGGAAGACTC
TCCTCCGTGCGTCCTCGTCTTCACCGGTCGCGTTCCTGAAACGCAGATGTGCCTCGCGCC
GCACTGCTCCGAACAATAAAGATTCTACAATACTAGCTTTTATGGTTATGAAGAGGAAAA
ATTGGCAGTAACCTGGCCCCACAAACCTTCAAATGAACGAATCAAATTAACAACCATAGG
ATGATAATGCGATTAGTTTTTTAGCCTTATTTCTGGGGTAATTAATCAGCGAAGCGATGAT
TTTTGATCTATTAACAGATATATAAATGCAAAAACTGCATAACCACTTTAACTAATACTTT
CAACATTTTCGGTTTGTATTACTTCTTATTCAAATGTAATAAAAGTATCAACAAAAAATTG
TTAATATACCTCTATACTTTAACGTCAAGGAGAAAAAACTATA

pADH1

GGGTGTACAATATGGACTTCCTCTTTTCTGGCAACCAAACCCATACATCGGGATTCCTATAA
TACCTTCGTTGGTCTCCCTAACATGTAGGTGGCGGAGGGGAGATATACAATAGAACAGATAC
CAGACAAGACATAATGGGCTAAACAAGACTACACCAATTACACTGCCTCATTGATGGTGGTA
CATAACGAACTAATACTGTAGCCCTAGACTTGATAGCCATCATCATATCGAAGTTTCACTACC
CTTTTTCCATTTGCCATCTATTGAAGTAATAATAGGCGCATGCAACTTCTTTTCTTTTTTTTT
CTTTTCTCTCTCCCCCGTTGTTGTCTCACCATATCCGCAATGACAAAAAAATGATGGAAGACA
CTAAAGGAAAAAATTAACGACAAAGACAGCACCAACAGATGTCGTTGTTCCAGAGCTGATGA
GGGGTATCTCGAAGCACACGAAACTTTTTCCTTCCTTCATTCACGCACACTACTCTCTAATGA
GCAACGGTATACGGCCTTCCTTCCAGTTACTTGAATTTGAAATAAAAAAAAGTTTGCTGTCTT
GCTATCAAGTATAAATAGACCTGCAATTATTAATCTTTTGTTTCCTCGTCATTGTTCTCGTTCC
CTTTCTTCCTTGTTTCTTTTTCTGCACAATATTTCAAGCTATACCAAGCATACAATCAACTATC
TCATATACA

pGPD

CAGTTCGAGTTTATCATTATCAATACTGCCATTTCAAAGAATACGTAAATAATTAATAGTAGTG
ATTTTCCTAACTTTATTTAGTCAAAAAATTAGCCTTTTAATTCTGCTGTAACCCGTACATGCCC
AAAATAGGGGGCGGGTTACACAGAATATATAACATCGTAGGTGTCTGGGTGAACAGTTTATTCC
TGGCATCCACTAAATATAATGGAGCCCGCTTTTTAAGCTGGCATCCAGAAAAAAAAAGAATCCC
AGCACCAAAATATTGTTTTCTTCACCAACCATCAGTTCATAGGTCCATTCTCTTAGCGCAACTAC
AGAGAACAGGGGCACAAACAGGCAAAAAACGGGCACAACCTCAATGGAGTGATGCAACCTGCCT
GGAGTAAATGATGACACAAGGCAATTGACCCACGCATGTATCTATCTCATTTTCTTACACCTTCTA
TTACCTTCTGCTCTCTCTGATTTGGAAAAAGCTGAAAAAAAAGGTTGAAACCAGTTCCCTGAAAT
TATTCCCCTACTTGACTAATAAGTATATAAAGACGGTAGGTATTGATTGTAATTCTGTAAATCTATT
TCTTAAACTTCTTAAATTCTACTTTTATAGTTAGTCTTTTTTTTAGTTTTAAAACACCAAGAACTT
AGTTTCGAATAAACACACATAAACAAACAAA

pTEF2

GCTACCTATATTCCACCATAACATCAATCATGCGGTTGCTGGTGTATTTACCAATAATGTTTAATGT
ATATATATATATATATATGGGGCCGTATACTTACATATAGTAGATGTCAAGCGTAGGCGCTTCCCCTG
CCGGCTGTGAGGGCGCCATAACCAAGGTATCTATAGACCGCCAATCAGCAAACTACCTCCGTACATT
CATGTTGCACCCACACATTTATACACCCAGACCGCGACAAATTACCCATAAGGTTGTTTGTGACGGC
GTCGTACAAGAGAACGTGGGAACTTTTTAGGCTCACCAAAAAAGAAAGAAAAAATACGAGTTGCT
GACAGAAGCCTCAAGAAAAAAAAAAATTCTTCTTCGACTATGCTGGAGGCAGAGATGATCGAGCCGG
TAGTTAACTATATATAGCTAAATTGGTTCCATCACCTTCTTTTCTGGTGTCGCTCCTTCTAGTGCTAT
TTCTGGCTTTTCCTATTTTTTTTTTTTCCATTTTTCTTTCTCTCTTTCTAATATATAAATTCTCTTGCA
TTTTCTATTTTTCTCTCTATCTATTCTACTTGTTTATTCCCTTCAAGGTTTTTTTTTAAGGAGTACTT
GTTTTTAGAATATACGGTCAACGAACTATAATTAACTAAAC

Coding Sequences

guide RNA

ATAAACTCATTTACTTATGTGTTTTAGAGCTAGAAATAGCAAGTTAAAATAAGGCTAGTCCGTT
ATCAACTTGAAAAAGTGGCACCGAGTCGGTGCTTTT

RGR

GTTTATCTGATGAGTCCGTGAGGACGAAACGAGTAAGCTCGTCATAAACTCATTTACTTATGTG
TTTTAGAGCTAGAAATAGCAAGTTAAAATAAGGCTAGTCCGTTATCAACTTGAAAAAGTGGCA
CCGAGTCGGTGCTTTTGGCCGGCATGGTCCCAGCCTCCTCGCTGGCGCCGGCTGGGCAACATG
CTTCGGCATGGCGAATGGGAC

yEGFP

ATGTCTAAAGGTGAAGAATTATTCACTGGTGTTGTCCCAATTTTGGTTGAATTAGATGGTGATGTTA
ATGGTCACAAATTTTCTGTCTCCGGTGAAGGTGAAGGTGATGCTACTTACGGTAAATTGACCTTAAA
ATTTATTTGTACTACTGGTAAATTGCCAGTTCCATGGCCAACCTTAGTCACTACTTTCGGTTATGGTG
TTCAATGTTTTGCGAGATACCCAGATCATATGAAACAACATGACTTTTTCAAGTCTGCCATGCCAGAA
GGTTATGTTCAAGAAAGAACTATTTTTTTCAAAGATGACGGTAACTACAAGACCAGAGCTGAAGTCAA
GTTTGAAGGTGATACCTTAGTTAATAGAATCGAATTAAAAGGTATTGATTTTAAAGAAGATGGTAACAT
TTTAGGTCACAAATTGGAATACAACTATAACTCTCACAATGTTTACATCATGGCTGACAAACAAAAGAA
TGGTATCAAAGTTAACTTCAAAATTAGACACAACATTGAAGATGGTTCTGTTCAATTAGCTGACCATTA
TCAACAAAATACTCCAATTGGTGATGGTCCAGTCTTGTTACCAGACAACCATTACTTATCCACTCAATCT
GCCTTATCCAAAGATCCAAACGAAAAGAGAGACCACATGGTCTTGTTAGAATTTGTTACTGCTGCTGGTA
TTACCCATGGTATGGATGAATTGTACAAATAA

NLS_dSpCas9

ATGTCTAGAGCCCCAAAGAAGAAGAGAAAAGTTAGACCCGGGGATAAGAAATACTCTATTGGTTTGGCTA
TCGGTACAAACTCTGTTGGTTGGGCTGTTATTACTGATGAATACAAGGTTCCATCCAAGAAGTTCAAGGT
TTTGGGTAACACTGATAGACACTCCATCAAAAAGAACTTGATTGGTGCCTTGTTGTTCGATTCTGGTGAA
ACTGCTGAAGCTACTAGATTGAAAAGAACCGCTAGAAGAAGATACACCAGAAGAAAGAACAGAATCTGCT
ACTTGCAAGAAATCTTCTCCAACGAAATGGCCAAGGTTGATGATTCATTCTTCCACAGATTGGAAGAATC
CTTCTTGGTCGAAGAAGATAAGAAGCACGAAAGACATCCAATCTTCGGTAACATCGTTGATGAAGTTGCT
TACCACGAAAAGTACCCAACTATCTACCATTTGAGAAAGAAGTTGGTTGACTCTACCGATAAGGCTGATTT
GAGATTGATCTATTTGGCTTTGGCCCACATGATTAAGTTCAGAGGTCATTTCTTGATCGAAGGTGATTTGA
ACCCAGATAACTCCGATGTTGATAAGTTGTTCATCCAATTAGTCCAAACCTACAATCAATTATTCGAAGAA
AACCCAATCAACGCCTCTGGTGTTGATGCTAAAGCTATTTTGTCTGCCAGATTGTCCAAGTCCAGAAGATT
AGAAAATTTGATCGCCCAATTACCAGGTGAAAAGAAGAATGGTTTGTTCGGTAATTTGATTGCCTTGTCTT
TGGGTTTGACTCCAAACTTCAAGTCCAATTTCGATTTGGCTGAAGATGCCAAGTTGCAATTATCTAAGGAT
ACCTACGATGACGATTTGGATAACTTGTTGGCTCAAATCGGTGATCAATACGCTGATTTGTTTTTGGCTGC
TAAGAACTTGTCCGATGCCATTTTGTTGTCCGATATTTTGAGAGTCAACACCGAAATTACTAAGGCTCCAT
TGTCTGCCTCTATGATCAAAAGATACGATGAACACCACCAAGACTTGACTTTGTTGAAGGCTTTGGTCAGA
CAACAATTACCTGAAAAGTACAAAGAAATTTTCTTCGATCAATCCAAGAACGGTTACGCCGGTTATATTGA
TGGTGGTGCTTCTCAAGAAGAATTTTACAAGTTCATCAAGCCAATCTTGGAAAAGATGGACGGTACTGAA
GAATTATTGGTCAAGTTGAACAGAGAAGATTTGTTGAGAAAGCAAAGAACCTTCGACAACGGTTCTATTC
CACATCAAATTCACTTGGGTGAATTGCACGCAATTTTGAGAAGACAAGAAGATTTTTATCCATTCTTGAA
GGACAACAGAGAAAAGATCGAAAAGATTCTGACCTTCAGAATCCCTTACTACGTTGGTCCATTGGCTAGAG
GTAATTCAAGATTTGCCTGGATGACTAGAAAGTCCGAAGAAACTATTACTCCTTGGAACTTCGAAGAAGTT
GTAGATAAGGGTGCTTCTGCCCAATCCTTTATTGAAAGAATGACCAACTTCGACAAGAACTTGCCAAACG
AAAAGGTTTTGCCAAAGCACTCTTTGTTGTACGAATACTTCACCGTCTACAACGAATTGACTAAGGTTAA
GTACGTCACCGAAGGTATGAGAAAACCAGCTTTTTTATCCGGTGAACAAAAGAAGGCTATCGTCGATTTG
TTGTTCAAGACCAACAGAAAGGTTACTGTCAAGCAATTAAAAGAAGATTACTTCAAGAAAATCGAATGCT
TCGACTCCGTTGAAATTTCTGGTGTCGAAGATAGATTCAATGCCTCTTTAGGTACTTACCATGACTTGTTG
AAAATCATCAAGGACAAGGATTTCTTGGACAACGAAGAAAACGAAGATATTTTGGAAGATATTGTCTTGA
CATTGACCTTGTTTGAAGATAGAGAAATGATTGAAGAAAGATTGAAAACCTACGCCCACTTGTTCGATGAT
AAGGTTATGAAGCAATTAAAGAGAAGAAGATACACTGGTTGGGGTAGATTGTCCAGAAAATTGATTAACG
GTATCAGAGACAAGCAATCCGGTAAGACCATTTTGGACTTTTTGAAGTCTGATGGTTTCGCTAACAGAAAC
TTCATGCAATTAATCCACGACGATTCCTTGACTTTCAAAGAAGATATACAAAAGGCCCAAGTCTCTGGTCA
AGGTGATTCTTTACATGAACATATCGCTAACTTGGCTGGTTCTCCAGCTATTAAGAAGGGTATTTTACAAA
CCGTTAAGGTCGTTGACGAATTGGTCAAAGTTATGGGTAGACATAAGCCAGAAAACATCGTTATCGAAATG
GCTAGAGAAAATCAAACCACCCAAAAGGGTCAAAAGAACTCCAGAGAAAGAATGAAGAGAATCGAAGAAG
GTATCAAAGAATTGGGTTCCCAAATTTTGAAAGAACACCCAGTTGAAAACACCCAATTACAAAACGAAAA
GTTGTACTTGTACTACTTGCAAAACGGTAGAGATATGTACGTTGACCAAGAATTGGACATCAACAGATTGT
CTGATTACGATGTTGACGCTATCGTTCCACAATCTTTTTTGAAGGATGACTCCATTGACAACAAGGTCTTG
ACTAGATCCGATAAGAATAGAGGTAAGTCCGATAACGTTCCATCTGAAGAAGTCGTTAAGAAAATGAAGAA
CTATTGGAGACAATTATTGAACGCCAAGTTGATCACCCAAAGAAAGTTTGACAATTTGACCAAGGCTGAAA
GAGGTGGTTTGTCTGAATTGGATAAGGCAGGTTTCATCAAAAGACAATTAGTAGAAACCAGACAAATCACC
AAGCACGTTGCTCAAATTTTGGATAGTAGAATGAACACTAAGTACGACGAAAACGACAAATTGATCAGAGA
AGTTAAGGTCATTACCTTGAAGTCCAAGTTGGTTTCCGATTTCAGAAAGGACTTCCAATTCTACAAGGTCA
GAGAAATCAACAACTACCATCATGCACATGATGCTTACTTGAATGCTGTTGTTGGTACTGCCTTGATTAAGA
AGTATCCAAAGTTGGAATCCGAATTTGTCTACGGTGATTACAAGGTTTACGACGTTAGAAAGATGATCGCCA
AGTCCGAACAAGAAATTGGTAAAGCTACTGCCAAATACTTCTTCTACTCCAATATTATGAATTTCTTTAAGA
CCGAAATCACTTTGGCCAACGGTGAAATTAGAAAAAGACCATTGATTGAAACTAATGGTGAAACAGGTGAA
ATCGTTTGGGATAAGGGTAGAGATTTTGCCACTGTTAGAAAGGTATTGTCCATGCCACAAGTAAACATCGTC
AAAAAGACCGAAGTTCAAACTGGTGGTTTCTCCAAAGAATCCATTTTGCCTAAGAGAAACTCCGATAAGTT
GATCGCTAGAAAAAAAGACTGGGACCCAAAAAAGTACGGTGGTTTTGATTCTCCAACTGTTGCTTACTCTG
TTTTGGTTGTTGCTAAGGTCGAAAAGGGTAAGAGTAAGAAGTTGAAGTCCGTCAAAGAATTATTAGGTATC
ACTATCATGGAAAGATCCTCATTCGAAAAGAATCCTATCGACTTTTTGGAAGCCAAGGGTTACAAAGAAGT
CAAGAAGGACTTGATCATTAAGTTGCCAAAGTACAGTTTGTTCGAATTGGAAAATGGTAGAAAGAGAATGT
TGGCTTCTGCCGGTGAATTACAAAAGGGTAATGAATTGGCTTTGCCATCCAAGTACGTTAATTTCTTATACT
TGGCCTCCCACTACGAAAAATTGAAAGGTTCTCCTGAAGATAACGAACAAAAGCAATTATTTGTCGAACAAC
ACAAGCACTACTTGGACGAAATCATTGAACAAATTTCCGAATTTTCCAAAAGAGTCATTTTGGCTGACGCCA
ATTTGGACAAAGTTTTGTCAGCTTACAACAAGCACAGAGATAAGCCAATTAGAGAACAAGCTGAAAACATC
ATTCACTTGTTCACTTTGACTAACTTGGGTGCTCCAGCTGCTTTTAAGTATTTCGATACCACTATCGACAGAA

AGAGATACACCTCTACCAAAGAAGTTTTGGACGCTACTTTGATCCACCAATCTATTACTGGTTTGTACGAAA
CTAGAATCGACTTGTCTCAATTAGGTGGTGATGGTTCTGGTAGATCTGGAGTCGACGGTGGATAA

Mxi1

ATGATTAATGTGCAAAGGCTGTTAGAAGCCGCAGAGTTTTTAGAAAGAAGAGAAAGAGAATGCGAACACG
GGTATGCCAGTTCTTTCCCTAGCATGCCCTCTCCCAGATGA

HBD(ER)

TCTGCTGGAGACATGAGAGCTGCCAACCTTTGGCCAAGCCCGCTCATGATCAAACGCTCTAAGAAGAACA
GCCTGGCCTTGTCCCTGACGGCCGACCAGATGGTCAGTGCCTTGTTGGATGCTGAGCCCCCCATACTCTAT
TCCGAGTATGATCCTACCAGACCCTTCAGTGAAGCTTCGATGATGGGCTTACTGACCAACCTGGCAGACAG
GGAGCTGGTTCACATGATCAACTGGGCGAAGAGGGTGCCAGGCTTTGTGGATTTGACCCTCCATGATCAG
GTCCACCTTCTAGAATGTGCCTGGCTAGAGATCCTGATGATTGGTCTCGTCTGGCGCTCCATGGAGCACCC
AGTGAAGCTACTGTTTGCTCCTAACTTGCTCTTGGACAGGAACCAGGGAAAATGTGTAGAGGGCATGGTG
GAGATCTTCGACATGCTGCTGGCTACATCATCTCGGTTCCGCATGATGAATCTGCAGGGAGAGGAGTTTGT
GTGCCTCAAATCTATTATTTTGCTTAATTCTGGAGTGTACACATTTCTGTCCAGCACCCTGAAGTCTCTGG
AAGAGAAGGACCATATCCACCGAGTCCTGGACAAGATCACAGACACTTTGATCCACCTGATGGCCAAGGC
AGGCCTGACCCTGCAGCAGCAGCACCAGCGGCTGGCCCAGCTCCTCCTCATCCTCTCCCACATCAGGCACA
TGAGTAACAAAGGCATGGAGCATCTGTACAGCATGAAGTGCAAGAACGTGGTGCCCCTCTATGACCTGCTG
CTGGAGATGCTGGACGCCCACCGCCTACATGCGCCCACTAGCCGTGGAGGGGCATCCGTGGAGGAGACGGA
CCAAAGCCACTTGGCCACTGCGGGCTCTACTTCATCGTAA

LmAcrIIA2

ATGACATTAACAAGAGCACAAAAAAAGTATGCAGAAGCGATGCACGAGTTTATAAATATGGTGGATGAC
TTTGAAGAGTCTACACCGGATTTTGCAAAGGAAGTTCTACATGATTCTGACTATGTAGTTATTACAAAA
AATGAAAAATATGCAGTAGCTCTTTGCTCTCTTAGCACTGATGAATGTGAATATGATACTAACTTATACT
TAGATGAAAAATTGGTTGATTACTCAACAGTTGACGTAAACGGTGTGACATACTACATCAACATTGTTGA
AACTAACGATATCGATGATTTAGAAATCGCTACGGATGAAGATGAGATGAAAAGTGGCAACCAAGAAATT
ATTTTAAAAAGTGAGTTGAAGTAA

LmAcrIIA4

ATGAATATTAATGACTTAATTAGAGAAATCAAAAACAAAGATTACACAGTGAAATTGAGTGGTACGGAT
AGCAATAGTATCACACAGCTAATTATTCGCGTTAATAATGATGGCAACGAGTATGTAATTTCTGAAAGT
GAAAATGAATCAATCGTTGAAAAATTCATCTCTGCATTCAAAAACGGTTGGAATCAAGAATACGAGGA
TGAAGAAGAATTTTATAATGACATGCAAACAATCACCTTAAAAAGTGAGTTGAACTAA

yo_LmAcrIIA4

ATGAATATTAATGATTTGATTAGAGAAATTAAAAATAAAGATTATACTGTTAAATTGTCTGGTACTGA
TTCTAATTCTATTACTCAATTGATTATTAGAGTTAATAATGATGGTAATGAATATGTTATTTCTGAATC
TGAAAATGAATCTATTGTTGAAAAATTTATTTCTGCTTTTAAAAATGGTTGGAATCAAGAATACGAA
GATGAAGAAGAATTTTATAATGATATGCAAACTATTACTTTGAAATCTGAATTGAATTAA

yo_StAcrIIA5

ATGGCTTATGGTAAATCTAGATATAATTCTTATAGAAAAAGATCTTTTAATAGATCTAATAAGCAAAG
AAGAGAGTACGCTCAGGAGATGGACAGGTTGGAGAAGGCTTTCGAGAACTTGGACGGTTGGTACTTG
TCTTCTATGAAAGATTCTGCTTATAAAGATTTTGGTAAATATGAAATTAGATTGTCTAATCATTCTGC
TGATAATAAATATCATGATTTGGAAAATGGTAGATTAATTGTTAATATTAAAGCTTCTAAATTGAATT
TTGTTGATATTATTGAAAATAAATTGGATAAAATTATTGAAAAAATTGATAAATTGGATTTGGATAAA
TACAGATTTATTAACGCTACTAATTTGGAACATGATATTAAATGTTATTATAAAGGTTTCAAAACTAAA
AAAGAAGTTATTTAA

Terminators

SUP4t

TTTTTTTGTTTTTTATGTCT

RPR1t

TTCTCGAGCCATATCCAACTTCCAATTTAATCTTTCTTTTTTAATTTTCACTTATTTGCGATACAGAA
AGAAAAAAGCGATAGTAACTATTGAATTTTGTTTGGATTTGGTTAGATTAGATATGGTTTCTCTTTA
TATTTACATGCTAAAAATGGGCTACACCAGAGATACATAATTAGATATATATACGCCAGTACACCTTAT
CGGCCCAAGCCTTGTCCCAAGGCAGCGTTTTGTTCTTGGAAACGCTGCCCTACACGTTCGCTATGCTT
CAAGAACTTTTCTGAGCACTTCATGATGCATGTTTGTTCCTTATTGGTTAGCTTTGATGTTGTGAAGT
CATTGACACAGTCTGTGAAACATCTTTCTACCAGATTAGAGTACAAACGCATGAAATCCTTCATTTGC
TTTTGTTCCACTACTTTTTGGAACTCTTGTTGTTCTTT

ADH1t

TTCTCGAGCCATATCCAACTTCCAATTTAATCTTTCTTTTTTAATTTTCACTTATTTGCGATACAGA
AAGAAAAAAGCGATAGTAACTATTGAATTTTGTTTGGATTTGGTTAGATTAGATATGGTTTCTCTT
TATATTTACATGCTAAAAATGGGCTACACCAGAGATACATAATTAGATATATATACGCCAGTACACCT
TATCGGCCCAAGCCTTGTCCCAAGGCAGCGTTTTGTTCTTGGAAACGCTGCCCTACACGTTCGCTAT
GCTTCAAGAACTTTTCTGAGCACTTCATGATGCATGTTTGTTCCTTATTGGTTAGCTTTGATGTTGT
GAAGTCATTGACACAGTCTGTGAAACATCTTTCTACCAGATTAGAGTACAAACGCATGAAATCCTT
CATTTGCTTTTGTTCCACTACTTTTTGGAACTCTTGTTGTTCTTT

CYC1t

CATGTAATTAGTTATGTCACGCTTACATTCACGCCCTCCCCCCACATCCGCTCTA
ACCGAAAAGGAAGGAGTTAGACAACCTGAAGTCTAGGTCCCTATTTATTTTTTTA
TAGTTATGTTAGTATTAAGAACGTTATTTATATTTCAAATTTTTCTTTTTTTTCTGTA
CAGACGCGTGTACGCATGTAACATTATACTGAAAACCTTGCTTGAGAAGGTTTTG
GGACGCTCGAAGGCTTTAATTTGCAAGCT
